# A role for vessel‐associated extracellular matrix proteins in multiple sclerosis pathology

**DOI:** 10.1111/bpa.13263

**Published:** 2024-04-25

**Authors:** Marco Pisa, Joseph L. Watson, Jonathan I. Spencer, Guy Niblett, Yasamin Mahjoub, Andrew Lockhart, Richard L. Yates, Sydney A. Yee, Gina Hadley, Jennifer Ruiz, Margaret M. Esiri, Benedict Kessler, Roman Fischer, Gabriele C. DeLuca

**Affiliations:** ^1^ Nuffield Department of Clinical Neurosciences University of Oxford Oxford UK; ^2^ Oxford Medical School University of Oxford Oxford UK; ^3^ Centre for Cardiovascular Medicine and Devices William Harvey Research Institute, Queen Mary University of London London UK; ^4^ Faculty of Medicine University of Alberta Edmonton Alberta Canada; ^5^ Mandell MS Center Trinity Health of New England Hartford Connecticut USA; ^6^ Mass Spectrometry Laboratory Target Discovery Institute, University of Oxford Oxford UK

**Keywords:** ECM proteins, HLA‐DRB1*15, MS, pathology, topography, vessel

## Abstract

Multiple sclerosis (MS) is unsurpassed for its clinical and pathological hetherogeneity, but the biological determinants of this variability are unknown. HLA‐DRB1*15, the main genetic risk factor for MS, influences the severity and distribution of MS pathology. This study set out to unravel the molecular determinants of the heterogeneity of MS pathology in relation to HLA‐DRB1*15 status. Shotgun proteomics from a discovery cohort of MS spinal cord samples segregated by HLA‐DRB*15 status revealed overexpression of the extracellular matrix (ECM) proteins, biglycan, decorin, and prolargin in HLA‐DRB*15‐positive cases, adding to established literature on a role of ECM proteins in MS pathology that has heretofore lacked systematic pathological validation. These findings informed a neuropathological characterisation of these proteins in a large autopsy cohort of 41 MS cases (18 HLA‐DRB1*15‐positive and 23 HLA‐DRB1*15‐negative), and seven non‐neurological controls on motor cortical, cervical and lumbar spinal cord tissue. Biglycan and decorin demonstrate a striking perivascular expression pattern in controls that is reduced in MS (−36.5%, *p* = 0.036 and − 24.7%, *p* = 0.039; respectively) in lesional and non‐lesional areas. A concomitant increase in diffuse parenchymal accumulation of biglycan and decorin is seen in MS (*p* = 0.015 and *p* = 0.001, respectively), particularly in HLA‐DRB1*15‐positive cases (*p* = 0.007 and *p* = 0.046, respectively). Prolargin shows a faint parenchymal pattern in controls that is markedly increased in MS cases where a perivascular deposition pattern is observed (motor cortex +97.5%, *p* = 0.001; cervical cord +49.1%, *p* = 0.016). Our findings point to ECM proteins and the vascular interface playing a central role in MS pathology within and outside the plaque area. As ECM proteins are known potent pro‐inflammatory molecules, their parenchymal accumulation may contribute to disease severity. This study brings to light novel factors that may contribute to the heterogeneity of the topographical variation of MS pathology.

## Graphical abstract

Shotgun proteomic and confirmatory immunohistochemical characterisation of post‐mortem tissue showed region‐specific and HLA‐DRB*15 influenced alteration in extracellular matrix proteins expression between MS and controls. In particular, we shows that non‐neurological controls have a perivascular‐predominant expression pattern of these proteins that is disrupted in MS where instead a marked parenchymal deposition pattern is seen. Our study provides unequivocal evidence for a role of extracellular matrix proteins and the vasculature being key contributors to MS pathology within and outside the plaque area.

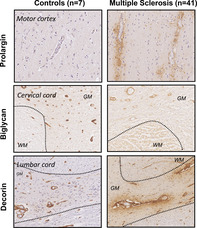

## 1 INTRODUCTION

Multiple scleriosis (MS) is unsurpassed for its heterogeneity in clinical and pathological outcome with both genetic and environmental factors playing important roles [1, 2, 3]. HLA‐DRB1*15 is the major MS genetic susceptibility allele, and associates with greater inflammatory activity, tissue loss and disability accrual in clinical cohort studies [4]. Work by our group and others has shown that MS cases carrying the HLA‐DRB1*15 allele are more inflammatory than their HLA‐DRB1*15‐negative counterparts [5, 6, 7, 8, 9]. Intriguingly, the extent to which HLA‐DRB1*15 influences the severity of pathology is not uniform throughout the CNS being exaggerated in the cervical spinal cord [5, 9].

The anatomy of the vasculature in the CNS, particularly the venular system, has long been seen as the culprit of MS pathology [10]. Early neuropathological descriptions furnished by Rindfleisch, Dawson, Fog, and Oppenheimer demonstrated anatomical relationships between lesion location and vascular supply [11, 12] which have been substantiated by subsequent pathological and radiographic studies [13, 14]. More recent work has extended these findings by showing that vascular pathology is a feature well beyond the lesional milieu and is associated with inflammation in “normal appearing” areas that likely contribute to disability accumulation [15]. The underlying biological substrate that drive these hallmark features of MS pathology and how HLA‐DRB1*15 status modulates them remain poorly understood.

We hypothesised that the heterogeneity of MS pathology is due to protein expression differences that are influenced by HLA‐DRB*15 status.

**FIGURE 1:**
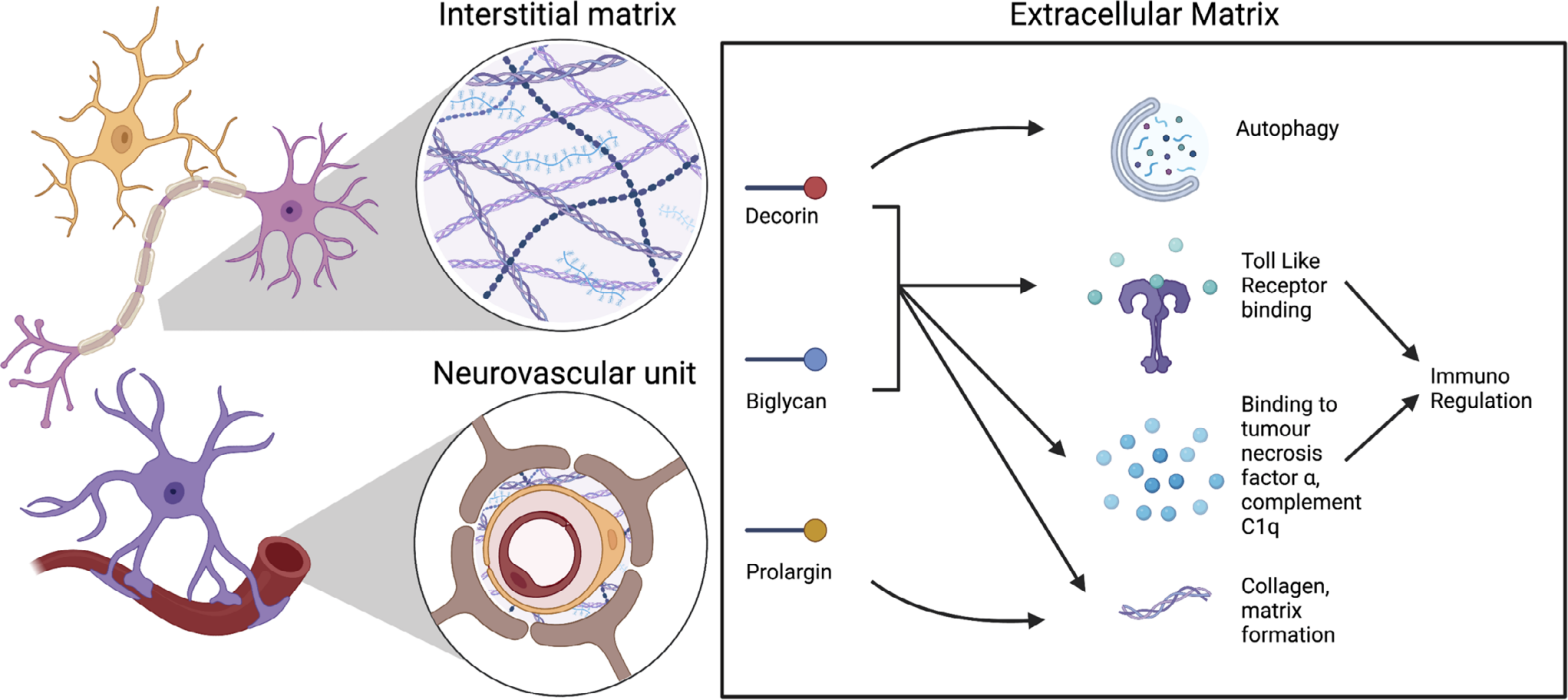
Functions of the ECM proteins decorin, biglycan and prolargin. Functions of the ECM proteins decorin, biglycan and prolargin. These three proteins are all part of the family of leucine‐rich proteoglycans. These proteins are expressed throughout the body. In the CNS these proteins can be found in the vascular basement membrane and in the interstitial matrix. Originally thought to be mainly important in bone growth, they have been found to be involved in a wide range of cellular functions. These include promoting autophagy (decorin), modulation of the immune system by interacting with toll like receptors and also cytokines such as tumour necrosis factor α, and in the formation of the ECM architecture [16, 26, 27, 28, 29, 30, 31, 48]. Created with BioRender.com.

A proof of concept shotgun proteomic interrogation of post‐mortem cervical spinal cord tissue derived from MS cases segregated by HLA‐DRB*15 status, identified three extracellular matrix (ECM) proteins, biglycan, decorin, and prolargin, that are overexpressed in HLA‐DRB1*15‐positive cases. Given the mounting evidence of these proteins as modulators of inflammation in MS (summarised in Figure 1 and reviewed elsewhere) [16], we conducted a systematic neuropathological characterisation of biglycan, decorin, and prolargin in a large human autopsy cohort of MS cases. In so doing, we revealed vessel‐associated expression of these ECM proteins in controls that is diffusely altered in MS cases. In MS, there is a striking accumulation of parenchymal expression of these proteins in compared to controls, particularly in HLA‐DRB1*15‐positive cases. Collectively, these findings highlight that ECM proteins and the vasculature are important contributors of pathological heterogeneity in progressive MS.

## 2 MATERIALS AND METHODS

### 2.1 Shotgun proteomics

To focus protein candidates relevant to the severity of MS pathology and the role of HLA‐DRB1*15 status in modulating their expression, discovery shotgun proteomics was undertaken on 1 mm fresh‐frozen cervical spinal cord tissue slices derived from five MS cases segregated by HLA‐DRB1*15 status (15+ *n* = 3; 15− *n* = 2). The cervical spinal cord was selected given its relevance to clinical outcome in MS and previous data showing that HLA‐DRB1*15 status exagerrated MS pathology at this level of the neuraxis. To minimise confounders that may contribute to pathological variability, cases from each genotype group were matched for lesion load and clinico‐demographic characteristics (Data S1A). Tissue was derived from the UK MS Tissue Bank in compliance with Human Tissue Act guidelines (REC 08/MRE09/31 + 5). Spinal cord specimens were subjected to nano liquid‐chromatography tandem mass spectrometry (LC–MS/MS) (Orbitrap Fusion Lumos) using optimised methods and workflow parameters (Data S1A) [17, 18].

## 3 IMMUNOHISTOCHEMICAL CHARACTERISATION OF PROTEIN HITS

### 3.1 Study cohort

A human autopsy cohort of pathologically confirmed MS cases (*n* = 41) from both HLA‐DRB1*15‐positive (*n* = 18) and HLA‐DRB1*15‐negative subgroups (*n* = 23), and non‐neurological control cases (*n* = 7) was obtained from the UK MS Tissue Bank in compliance with Human Tissue Act guidelines (REC 08/MRE09/31 + 5). Three levels of the neuraxis relevant for disability progression, namely motor cortex, cervical and lumbar spinal cord, were available for each case.

### 3.2 Experimental procedure

Formalin‐fixed paraffin‐embedded 6 μm‐thick adjacent sections from each case and level of the neuraxis were immunostained using primary antibodies for myelin (PLP, BioRad, # MCA839G), prolargin (PRELP, Abcam, #ab135595), decorin (DEC, Millipore, #MAB143), biglycan (BGN, Invitrogen, #PA5‐82066) (Data S1B). Omission of primary antibody was used as negative controls (Data S1C).

### 3.3 Immunohistological analyses

A systematic qualitative assessment of the expression of top proteomic hits (biglycan, decorin, and prolargin) was conducted in human post‐mortem MS and control tissue by two independent assessors (GD and MP). Qualitative observations were recorded in parallel, and concordant observations are reported in the manuscript together with representative images. Based on the characteristic immunoreactivity patterns of each protein hit, we designed a tailored quantitative assessment, as outlined below.

All slides were scanned using the Aperio ScanScope at ×200 magnification. For each case, PLP‐stained images were used to guide delineation of regions of non‐lesional white and grey matter and lesional white and grey matter. An adjacent section was stained for CD68 to allow classification of each spinal cord plaque into active, mixed active‐inactive and chronic inactive, based on criteria from Kuhlmann and colleagues [19].

#### 3.3.1 Decorin and biglycan

Decorin and biglycan demonstrated two distinct staining patterns in the spinal cord: (1) perivascular staining which was quantified using an automated method; and (2) parenchymal staining for which a semiquantitative scoring method was applied. Perivascular co‐expression of decorin (R&D Systems, #MAB143) and biglycan (ThermoFisher, #PA5‐82066) was evaluated by double‐labelled immunohistochemistry (Figure 3). The perivascular expression of decorin and biglycan proteins prompted immunolabelling of sections with a cocktail of endothelial markers (CD31, Dako, #M0823; CD34, Serotec, # MCAP547) to allow analysis of vascular coverage (see below). The paucity of perivascular and parenchymal staining of decorin and biglycan in the motor cortex precluded quantitative and semiquantitative analyses in this region.

All biglycan and decorin immunolabelled images were exported to Fiji [20]. RGB images were binarised and perivascular biglycan or decorin was selectively detected and quantified using an optimised macro. This allowed systematic selection of binarised vessels and their corresponding lumen. Vessels were only selected if cut in the transverse plane enclosing a single, unstained lumen. An area index (AI) was determined for each vessel, by taking the extent of perivascular staining and controlling for the size of the corresponding lumen (Data S1D).

Assessment of parenchymal biglycan and decorin staining was performed using a semiquantitative score validated by two observers (Joseph L. Watson and Gabriele C. DeLuca) and ranged from 0, where no extracellular deposition was seen, to 2, where accumulation was intense (Data S1E).

#### 3.3.2 Prolargin

Prolargin demonstrated diffuse parenchymal immunoreactivity prompting use of a quantitative method for positive pixel detection on the whole sampled area in the motor cortex and cervical and lumbar spinal cord.

Prolargin immunolabelled images were analysed in ImageScope v12.1, where a pixel count for positive and negative pixels was undertaken using an optimised Aperio pixel count algorithm. The number of chromogen positive pixels/mm^2^ was determined for each region.

#### 3.3.3 Vascular coverage

Given the predominant perivascular expression of decorin and biglycan in the spinal cord, a systematic analysis of vascular coverage was undertaken. Sections were stained for CD31/CD34 and imaged in ImageScope. Vascular coverage was calculated using Fiji Analyze Particles tool on pre‐specified areas of the spinal cord, in both the lesional and non‐lesional tissue (Data S1F) [5].

Double‐labelled immunohistochemistry was used to assess whether perivascular changes in ECM protein expression were affecting arterioles, venules or both. Smooth muscle actin was used to visualise arterioles. Given that decorin and biglycan showed co‐localisation around vessels, co‐expression of biglycan and prolargin with smooth muscle actin (Dako, #M0851) were evaluated by double‐labelled immunohistochemistry (Data S5H and S7E).

### 3.4 Statistical analyses

Statistical analyses were performed using SPSS version 26 (SPSS, Chicago, IL). Generalised Estimating Equation linear (GEE) models were built considering biglycan area index, decorin area index and prolargin pixel count as dependent variables in order to take into account random subject‐related variability. Disease status (MS vs. controls), HLA status (HLA‐DRB1*15 positive vs. negative), Site (cortex, lumbar and cervical spinal cord), Matter type (gray vs. white matter) and Lesion status (lesional vs. non‐lesional) were considered in the models as independent factors together with their interaction terms. Possible factorial analyses were limited to those selected in a predefined pipeline (Data S1G). The association of parenchymal distribution of biglycan and decorin with Disease status (MS vs. controls), HLA status (HLA‐DRB1*15 positive vs. negative), and site (cortex, lumbar and cervical spinal cord area) was assessed using cross‐tab analyses. For all analyses, statistical significance were set at *p* < 0.05. Graphs were drawn using GraphPad Prism version 9.2 (GraphPad Software, San Diego, CA), with all bars illustrated as mean ± SEM.

## 4 RESULTS

### 4.1 Proteomic analysis

Discovery shotgun proteomics identified 1656 protein groups of which 804 were quantified with two peptides or more with 99 identified proteins differing between genotype groups (*p* < 0.01). The top three differentially expressed proteins between HLA‐DRB1*15 subgroups were small‐leucine rich proteoglycan ECM proteins, namely decorin, biglycan and prolargin, which were all over‐expressed in HLA‐DRB1*15‐positive cases (Data S2A,B). Further neuropathological characterisation of these proteins was undertaken in light of these findings and their biological relevance to immune modulation in MS.

### 4.2 Neuropathological validation of biglycan, decorin, and prolargin expression

Cohort characteristics of the studied MS (*n* = 41; 15+ *n* = 18, 15− *n* = 23) and non‐neurological controls (*n* = 7) cases are reported in Table 1. Non‐neurological controls and HLA‐DRB1*15‐positive and ‐negative MS cases were matched for sex, brain weight, post‐mortem interval (p.m.i.) and disease duration. Age at death was higher in non‐neurological controls compared with the two MS subgroups (*p* = 0.02). HLA‐DRB1*15‐positive cases tended to display more frequent demyelination, proportionally larger areas of demyelination, and higher frequency of active lesions compared to HLA‐DRB1*15‐negative (Data S3A). Lymphocytic CD3+, CD8+ and CD20+ inflammation was also numerically higher in HLA‐DRB1*15‐positive cases (Data S3B).

**TABLE 1:** Cohort characteristics.

	Multiple sclerosis HLA‐DRB1*15‐positive (*n* = 18)	Multiple sclerosis HLA‐DRB1*15‐negative (*n* = 23)	Non‐neurological controls (*n* = 7)
Age (years)	65.33 (range 40–92)	61.17 (range 43–82)	76.86 (range 64–91)
Sex	11 female (7 male)	17 female (6 male)	4 female (3 male)
Brain weight (grams)	1164 (range 1000–1364)	1162 (range 935–1380)	1249 (range 1090–1465)
P.m.i. (hours)	19.2 (range 7–38)	17.9 (range 7.5–28)	25.2 (range 10–48)
Disease duration (years)	34.2 (range 16–58)	28.6 (range 11–50)	N/A

**FIGURE 2:**
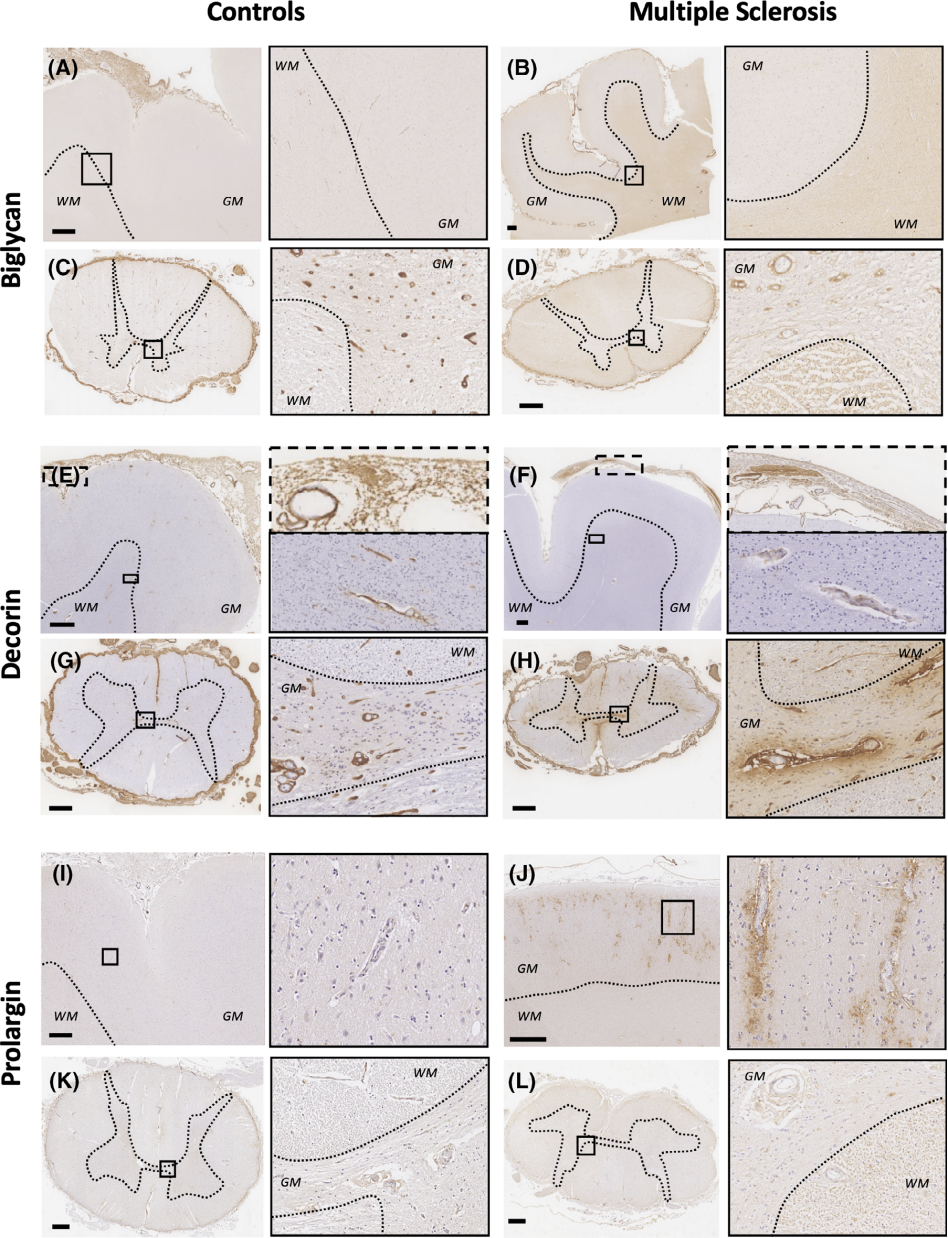
Immunoreactivity of biglycan, decorin, and prolargin in MS and controls. Biglycan staining in controls (A, C) and MS (B, D). In the motor cortex, biglycan shows minimal subcortical white matter deposition in controls (A) that contrasts with MS where an intense diffuse parenchymal staining pattern is observed (B). In the spinal cord, controls demonstrates a clear perivascular staining pattern (C, inset) that is markedly reduced in MS (D, inset) where a concomitant diffuse parenchymal staining is observed, particularly in white matter. Decorin staining in controls (E, G) and MS (F, H). In the motor cortex, decorin is homogeneously expressed in the meninges (E, top inset) and parenchymal perivascular spaces (E, bottom inset) of controls. In comparison, MS shows reduced decorin expression in the meninges (where it is limited to the adventitia of meningeal pial vessels) (F, top inset) and in parenchymal perivascular areas (F, bottom inset). In the spinal cord, controls show prominent perivascular and minimal parenchymal staining (G), which differs markedly in MS where reduced perivascular staining and increased diffuse parenchymal expression abutting Virchow Robin spaces (H) are seen. Prolargin staining in controls (I, K) and MS (J, L). In the motor cortex, controls show minimal prolargin staining (I), which contrasts significantly with MS where striking deposition is seen in the parenchyma abutting the Virchow Robin spaces, especially in the subpial and infragranular areas (J). In the spinal cord, controls have sparse staining (K) compared to MS case, which demonstrate a diffuse, faint parenchymal staining pattern in white matter with relative sparing of grey matter (L). The scale bar corresponds to 1 mm. GM, grey matter; WM, white matter.

**FIGURE 3:**
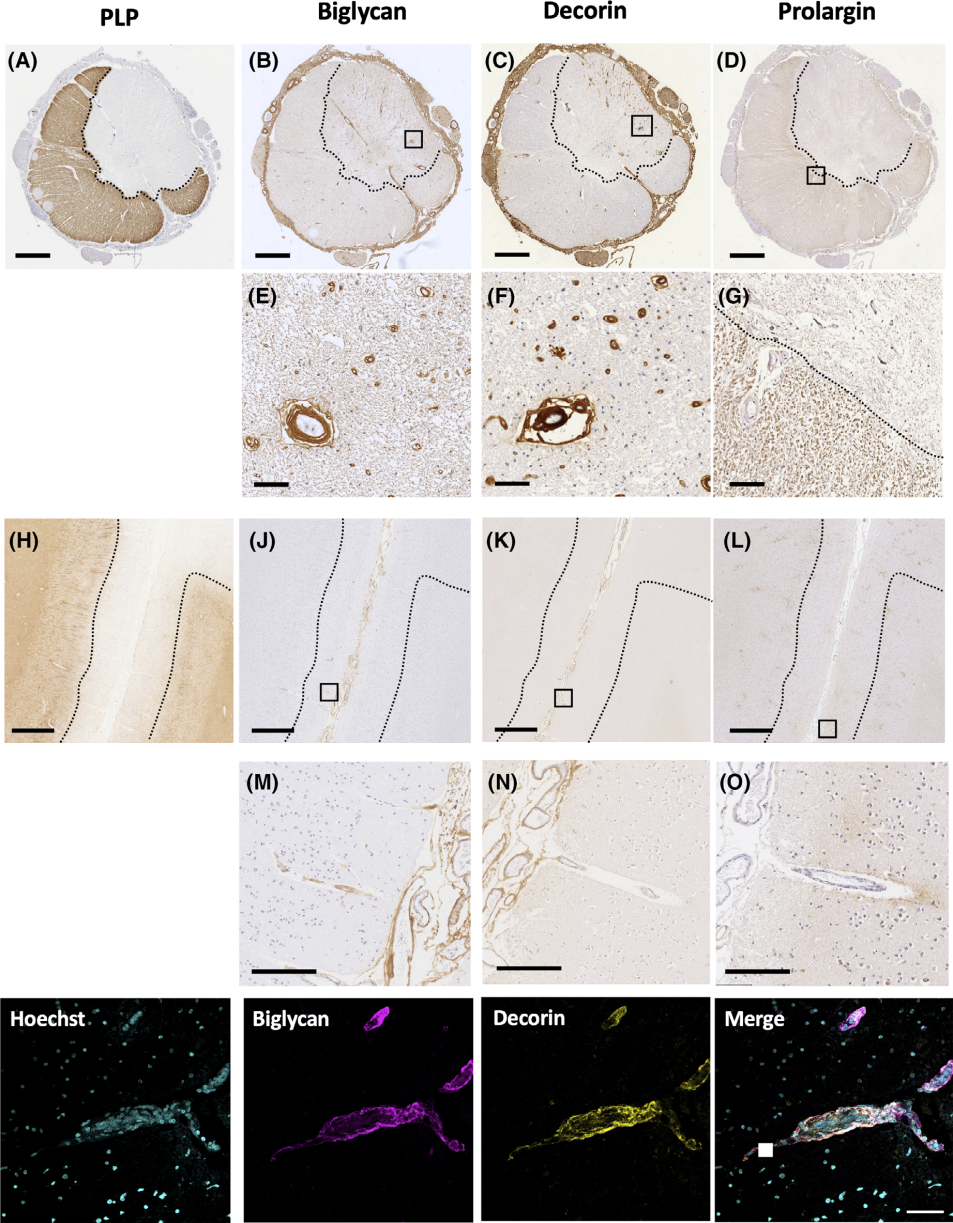
Immunoreactivity of biglycan, decorin, and prolargin along the neuraxis. Cervical spinal cord (A–G) and motor cortex (H–O) from a prototypical MS case immunolabelled for myelin (PLP) (A, H), biglycan (B, E, J, M), decorin (C, F, K, N), and prolargin (D, G, L, O). Well circumscribed areas of demyelination are seen in the spinal cord (A) and subpial cortex (H) (demarcated by dashed lines in A–D and H–L). In the spinal cord, biglycan (B) and decorin (C) show meningeal staining in the adventitia of pial vessels with an intense perivascular expression pattern within the Virchow Robin spaces of parenchymal vessels, particularly within lesional areas (E, F). Motor cortex shows reduced meningeal expression and minimal cortical parenchymal expression of biglycan (J) and decorin (K). Double immunofluorescence labelling of biglycan and decorin show co‐localisation of these proteins (bottom row images). Prolargin expression is diffuse throughout the spinal cord parenchyma, particularly in non‐lesional areas (D, G). In the motor cortex, prolargin demonstrates increased expression in the parenchyma abutting the Virchow Robin space (L, O), notably in subpial and infragranular cortical areas. Scale bars correspond to 1 mm in A–D and H–L, 50 μm in E–G and M–O.

Biglycan, decorin and prolargin expression showed MS‐specific topographical differences along the three levels of the neuraxis influenced by HLA‐DRB1*15 status (Figures 2 and 3).

### 4.3 Qualitative observations

#### 4.3.1 Biglycan

Biglycan was diffusely and intensely expressed in the meninges, including the adventitia of arterioles and venules in motor cortex and spinal cord in both MS and controls (Figures 2 and 3).

In the motor cortex, sparse perivascular biglycan immunoreactivity labelling the vascular adventitia within the Virchow‐Robin space (named perivascular hereafter) was predominantly found in the most superficial cortical layers and in the deep subcortical white matter both in controls and MS cases. Diffuse extracellular expression of biglycan in the parenchyma was variably observed in the white matter, predominantly in MS cases (Figure 2A,B).

In the spinal cord of control cases, perivascular biglycan staining was consistently seen in arterioles and venules throughout the parenchyma. Faint extracellular parenchymal biglycan staining pattern with relative sparing of the central canal and grey matter was also occasionally observed. In MS cases, the spinal cord showed marked reduction of perivascular biglycan expression in arterioles and venules, a finding that was variable between MS cases. Lesional areas occasionally showed increased perivascular expression compared with non‐lesional areas. In contrast, biglycan extracellular staining was clearly increased throughout the MS spinal cord parenchyma, predominantly in white matter (Figures 2C, D and 3).

A variable degree of glial expression of biglycan was seen in the subpial layers of both motor cortex and spinal cord in both MS and controls (Figure S5A).

#### 4.3.2 Decorin

Decorin diffusely stained the meninges, including the adventitia of blood vessels in the motor cortex and spinal cord. In some MS cases, however, meningeal expression was patchy and restricted to the perivascular areas only (Figures 2 and 3).

In the motor cortex, controls showed a perivascular pattern of decorin immunoreactivity in subpial and deep subcortical white matter vessels that was reduced in frequency and intensity in MS (Figure 2E, F).

In the spinal cord, controls showed intense perivascular decorin expression in the parenchyma. Diffuse extracellular expression of decorin was occasionally observed in a minority of cases with more abundant accumulation outside the Virchow Robin space seen in a small number of vessels. In MS, perivascular staining was markedly reduced compared with controls and variably so between MS cases. Further, within MS cases, perivascular staining was more consistently expressed in lesional areas compared to non‐lesional ones (Figure 3). Spinal cord extracellular parenchymal staining was clearly increased in MS cases, predominantly surrounding vessels, with confluence occasionally seen in both grey and white matter (Figure 2G, H).

#### 4.3.3 Prolargin

Faint prolargin immunoreactivity was found in the meninges with accentuation in the vascular adventitia in both motor cortex and spinal cord (Figures 2 and 3).

In motor cortex, controls exhibited extremely faint prolargin expression throughout the grey matter parenchyma. In contrast, MS cases consistently showed striking parenchymal prolargin immunoreactivity surrounding arterioles and venules outside the Virchow Robin space with variable expression of intensity between MS cases. This vessel‐associated expression pattern was present in the parenchyma of all cortical layers with accentuation in the subpial zone (especially in the depths of sulci) and in infragranular layers. A granular staining pattern was also observed in the parenchyma in what appeared to be within neuronal and glial cytoplasm (Figure 2I, J, Data S7A).

In the spinal cord, controls showed a faint perivascular pattern of prolargin expression together with parenchymal extracellular myelin‐associated staining. Absent prolargin staining was variably observed in the central canal and grey matter. In MS, in non‐lesional areas, a similar but more intense extracellular parenchymal staining pattern was seen. Lesional areas were devoid of prolargin staining (Figures 2K, L and 3).

Glial expression was observed in a subset of cases, particularly in cortical grey matter where parenchymal staining was most intense but also in the spinal cord in both MS and control cases.

### 4.4 Quantitative neuropathological analysis of vascular area, decorin, biglycan and prolargin

The results of qualitative analyses instructed a tailored quantitative neuropathological assessment as outlined in the methods. In brief, for biglycan and decorin, quantification aimed at understanding the differences in the intensity of perivascular and parenchymal staining in the cervical and lumbar cord. For prolargin, overall immunoreactivity in cortical and spinal cord tissues was quantified. Given the expression of biglycan and decorin within the Virchow Robin space, proportional vascular area was also calculated and used as a covariate in quantitative analyses of these ECM proteins.

#### 4.4.1 Vascular area

**FIGURE 4:**
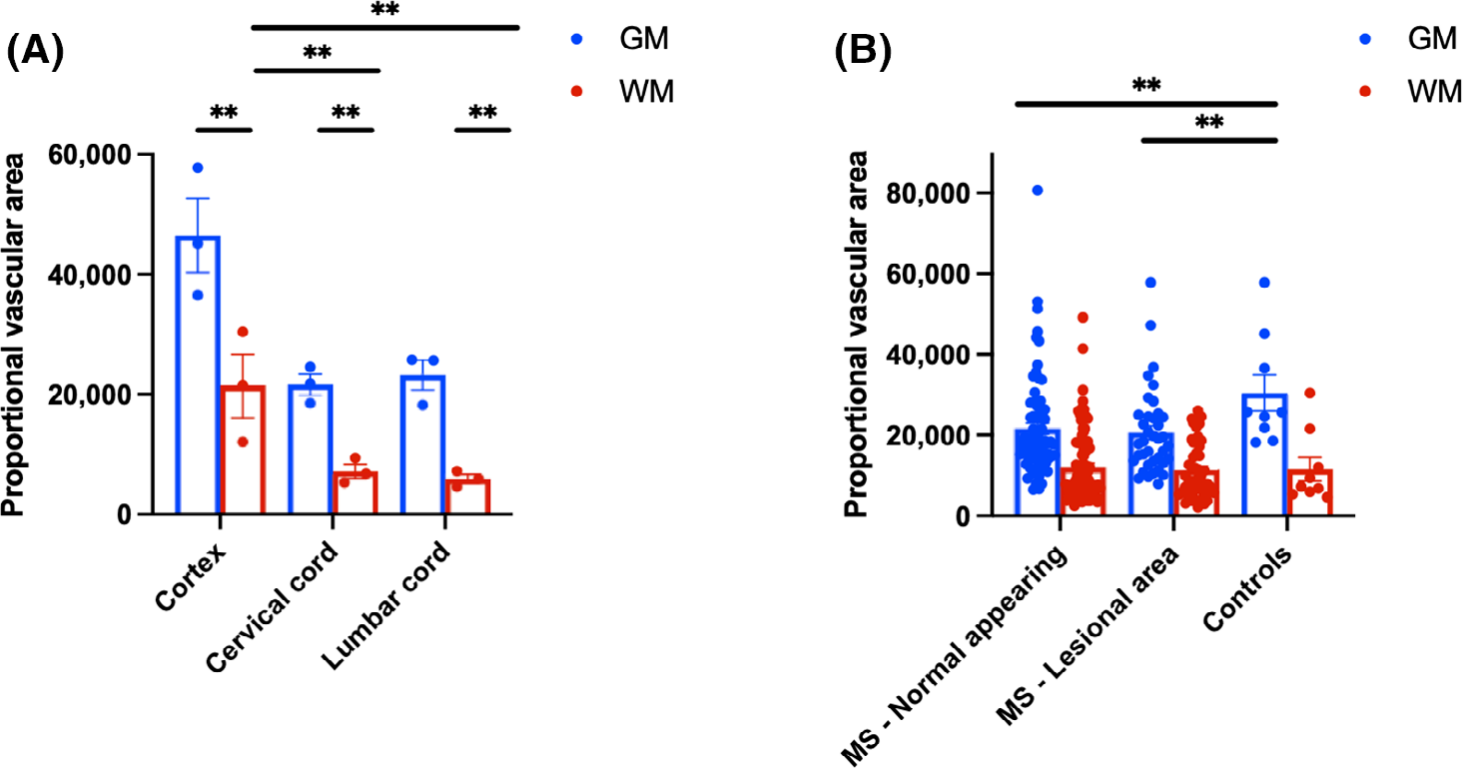
Topographical distribution of vascular area in MS cases and controls. (A) Vascular area in non‐neurological controls (*n* = 3) is presented according to matter type (white and grey) and site (cortex, cervical and lumbar spinal cord). Total vascular area is higher in grey compared to white matter throughout the neuraxis, and in the primary motor cortex compared to cervical and lumbar levels of the spinal cord. (B) Vascular area in lesional and non‐lesional areas in MS (*n* = 39) and non‐neurological controls (*n* = 3) according to matter type. Vascular area is greater in grey matter compared to white matter in each area type with grey matter vascularity being reduced in MS tissue compared to controls. Bars illustrate mean ± SEM. Relevant corrected Wald pairwise comparisons are displayed: **p < 0.001; *p < 0.05.

In controls, grey matter had nearly three times more vascular area than white matter (Matter type effect: *X* ^2^ 474.2, *p* < 0.001), consistent with previous reports [21]. The motor cortical grey and subcortical white matter areas also had higher vascularity compared to the cervical or lumbar spinal cord (Site effect: *X* ^2^ 366.6, *p* < 0.001), particularly in white matter (Matter type*Site effect: *X* ^2^ 8.9, *p* = 0.012; Figure 4, Data S4A).

MS cases exhibited reduced vascular area in both lesioned (−27%, *p* < 0.001) and non‐lesioned areas compared to controls (−20.26%, *p* = 0.005; Disease status*Lesional status: *X* ^2^ 13.73, *p* = 0.001). The difference between MS and controls was primarily driven by reduced vasculature in grey matter when analysing grey and white matter separately (Figure 4, Data S4B). Among MS cases, there were no differences related to HLA‐DRB1*15 genotype groups, site, or matter type interactions (Data S4C).

#### 4.4.2 Biglycan

**FIGURE 5:**
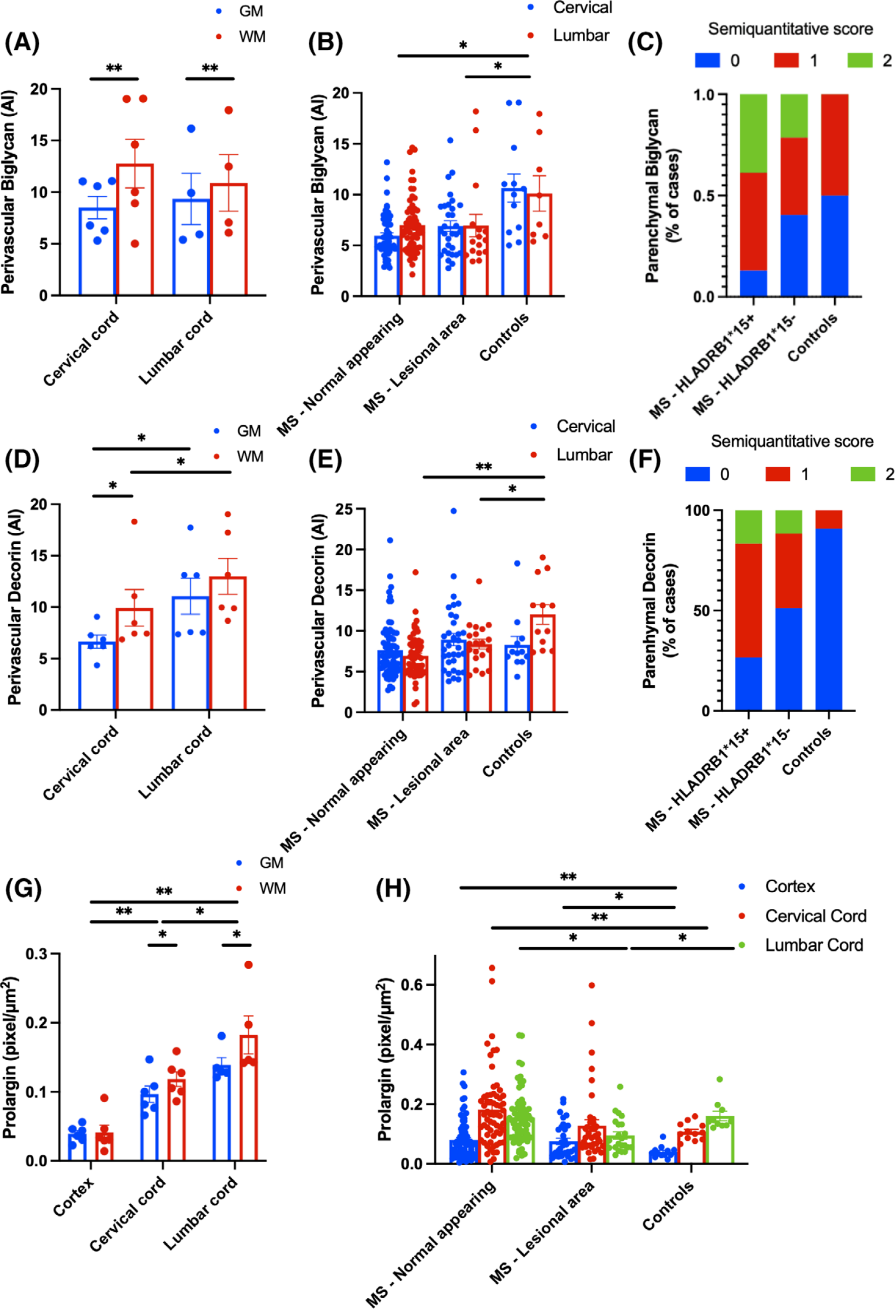
Topographical distribution of biglycan, decorin and prolargin expression in controls and MS. Biglycan (A–C), decorin (D–F), and prolargin (G, H) expression in controls and MS. (A) In controls, perivascular biglycan expression is increased in white compared to grey matter, particularly at cervical level. (B) In MS, perivascular biglycan expression is reduced compared to controls, regardless of spinal cord level or lesional status. In contrast, parenchymal biglycan expression (C) is increased in MS compared with controls, particularly in cases harbouring the HLA‐DRB1*15 allele. (D) In controls, perivascular decorin expression is increased in white compared with grey matter and in the lumbar compared with cervical cord levels. (E) Perivascular decorin expression is reduced in lesional and non‐lesional MS compared with controls at the lumbar cord level only. (F) In contrast, parenchymal decorin expression is increased in MS compared with controls, particularly in cases harboring the HLA‐DRB1*15 allele. (G) In controls, prolargin shows a gradient of expression along the neuraxis, being lowest in the cortex and highest in the lumbar cord. Within the cervical and lumbar cord, prolargin immunoreactivity is greater in white compared with grey matter. (H) Prolargin expression is increased in MS compared with controls in the cortex and cervical cord, particularly in non‐lesional white matter areas. Bars illustrate mean ± SEM. Relevant Wald pairwise comparisons are displayed: **p < 0.001; *p < 0.05. AI, area index.

In controls, white matter exhibited 32.5% higher perivascular biglycan immunoreactivity compared to grey matter (Matter type: *X* ^2^ 14.13, *p* < 0.001). The difference between white and grey matter was more pronounced in the cervical cord (+50.1%) than in the lumbar cord (+16.6%, Site*Matter type: *X* ^2^ 5.14, *p* = 0.023) (Figure 5A, Data S5B).

Overall, MS cases had reduced vascular but higher parenchymal expression of biglycan compared to controls regardless of lesion status.

MS cases displayed a 36.5% reduction in perivascular biglycan expression compared to controls (Disease status: *X* ^2^ 4.4, *p* = 0.036; Figure 5B, Data S5C). This reduction was consistent across spinal cord levels, with no effect of Site or Disease status*Site interaction. However, when adjusting for proportional vascular area, the biglycan loss in MS cases trended towards being more pronounced at the cervical cord level (−35.7% compared with controls, *p* = 0.076) than at the lumbar cord level (−12.8%, *p* > 0.1; Disease statusSite: *X* ^2^ 10.35, *p* = 0.001). This effect was confirmed when considering grey and white matter separately (Disease statusSite: *X* ^2^ 4.26, *p* = 0.039 for grey matter; *X* ^2^ 12.96, *p* < 0.001 for white matter). No difference was observed between lesional and non‐lesional MS tissue (no Lesion status effect or Lesion status*Disease status interaction). When considering demyelinated areas, active lesions showed lower perivascular biglycan compared to mixed active‐inactive and inactive lesions (Data S5F).

No differences were found in perivascular biglycan levels between HLA subgroups within MS cases, and there was no interaction between HLA status and anatomical site, matter type, or lesion staging (Data S5D–E).

In contrast, parenchymal biglycan was increased in MS cases compared to controls (Figure 5C). The majority of HLA‐DRB1*15‐positive cases showed an 80% increased probability of intense parenchymal staining (*p* = 0.007) (Data S5G). There was no association between parenchymal biglycan immunoreactivity and the anatomical site (cervical vs. lumbar cord). Parenchymal biglycan immunoreactivity was not associated with CD3+, CD8+ or CD20+ lymphocytic or CD68+ microglia‐macrophage inflammation.

#### 4.4.3 Decorin

In controls, perivascular decorin was higher in white matter than grey matter (Matter type: *X* ^2^ 6.89, *p* = 0.009) and in the lumbar cord compared to the cervical cord (Site: *X* ^2^ 11.76, *p* = 0.001), with no Site and Matter type interaction (Figure 5D, Data S6A).

Similar to biglycan, MS cases had reduced vascular but higher parenchymal expression of decorin compared to controls regardless of lesion status.

MS cases had a 24.7% reduction in perivascular decorin compared to controls (Disease status: *X* ^2^ 4.26, *p* = 0.039), driven mainly by the lumbar cord (−39.4%, *p* = 0.002). No significant change in cervical cord decorin was observed between MS and controls (−2.8%, *p* > 0.1; Disease status*Site: *X* ^2^ 16.01, *p* = 0.001; Figure 5E). The lumbar‐specific decorin reduction was confirmed for both grey (*X* ^2^ 9.74, *p* = 0.002) and white matter (*X* ^2^ 9.3, *p* = 0.002). A more pronounced reduction occurred in non‐lesional areas (−28.15%, *p* = 0.016; Disease status*Lesion status: *X* ^2^ 14.67, *p* = 0.001) compared to lesional areas (−14.1%, *p* > 0.1) compared to controls (Figure 5E, Data S6B). When considering demyelinated areas, active lesions showed lower perivascular decorin compared to mixed active‐inactive and inactive lesions (Data S6E).

HLA‐DRB1*15 status did not impact decorin levels in MS cases or interact with Site, Matter type, or lesion stage (Data S6C,D).

Similarly, parenchymal decorin was increased in MS cases compared to controls (Figure 5F, Data S6F). Controls rarely showed parenchymal decorin (only 1 of 11 control cord levels displayed mild staining), while 58.9% of MS cases had parenchymal decorin, with 13.7% showing intense staining (*p* = 0.001). Parenchymal decorin staining was more frequent in MS cases with the HLA‐DRB1*15‐positive allele (73.4%) compared to those without it (48.8%, *p* = 0.028). HLA‐DRB1*15‐positive cases also had a 50% increased probability of intense parenchymal staining (*p* = 0.046; Data S6F). No association was found between parenchymal decorin and the anatomical site (cervical vs. lumbar cord).

Parenchymal decorin immunoreactivity was not associated with CD3+, CD8+ or CD20+ lymphocytic or CD68+ microglia‐macrophage inflammation.

#### 4.4.4 Prolargin

In controls, prolargin levels varied along the neuraxis, being lowest in the motor cortex and highest in the lumbar cord (+300% compared to cortex, *p* < 0.001, and +50% compared to cervical cord, *p* = 0.01; Site: *X* ^2^ 64.51, *p* < 0.001; Figure 5G, Data S7B). Prolargin was 22.2% higher in white matter than grey matter (Matter type: *X* ^2^ 15.55, *p* < 0.001), with no Site*Matter type interaction.

In MS cases, prolargin increased in the cortex (+97.5%, *p* = 0.001) and cervical cord (+49.1%, *p* = 0.016) compared to controls, while no difference was seen in the lumbar cord (+6%, *p* > 0.1; Disease status*Site: *X* ^2^ 96.6, *p* < 0.001; Figure 5H, Data S7C). This increase was confirmed in both grey (*X* ^2^ 113.5, *p* < 0.001) and white matter (*X* ^2^ 76.4, *p* < 0.001). Prolargin mostly increased in non‐lesional areas in MS (+37.4%, *p* = 0.01), with no change in lesional areas (+3%, *p* > 0.1; Disease status*Lesion status: *X* ^2^ 14.02, *p* = 0.001). No difference in prolargin expression was found between lesion stages or HLA‐DRB1*15 genotype groups in MS cases, and there was no interaction with site or matter type. No interaction was observed when considering lesional and non‐lesional areas separately (Data S7D–F).

## 5 DISCUSSION

This study set out to unravel the determinants of heterogeneity of MS pathology throughout the neuraxis. Our discovery shotgun proteomic approach revealed overrepresentation of the ECM proteins, biglycan, decorin, and prolargin, in HLA‐DRB1*15 MS cases prompting neuropathological characterisation of these proteins. Through the use of a large human autopsy cohort of non‐neurological controls and MS cases, we demonstrated that vessel‐associated expression of these ECM proteins was markedly altered in MS regardless of lesion status. In particular, expression of these proteins was reduced in the Virchow Robin space and increased in the parenchyma with modulation by the HLA‐DRB1*15 allele in MS compared to controls. Given the immunomodulatory role of these ECM proteins in MS, these findings provide further compelling evidence that ECM proteins and the vasculature have important roles in MS pathogenesis and contribute to its pathological heterogeneity throughout the neuraxis.

### 5.1 Perivascular ECM protein expression is reduced in MS compared to controls reveal diffuse vessel‐associated pathology in MS regardless of lesional status

ECM protein expression is altered around blood vessels in MS. In controls, biglycan and decorin are predominantly expressed within the perivascular space with a striking reduction of vessel‐associated expression in MS. There is a concomitant increased expression of decorin and biglycan in the parenchyma both within and outside of lesions in MS compared to controls. Similarly, prolargin expression, largely absent in controls, shows a striking parenchymal deposition around vessels in MS. Strikingly, the vessel‐associated protein changes affect both arterioles and venules even outside of lesions. Moreover, vascular density is reduced in MS compared to controls both within and outside of lesions. The alterations in vascular density and ECM proteins point to the vasculature as a central culprit of MS pathology even outside demyelinated lesions.

Historically, the dogma that MS pathology centres on the demyelinated lesion surrounding venules has predominated the literature [12, 22]. Only recently, pathology outside lesions and beyond the venous system has gathered attention [13, 15, 23, 24, 25] Periarterial small vessel disease outside of lesions associates with inflammatory disease activity in MS [13]. Further, fibrinogen deposition, a surrogate marker of blood–brain barrier disruption, is extensive outside the plaque and relates to neurodegeneration [24]. Radiological data show reduced perfusion in the cerebral cortex and abnormal diffusion along the perivascular space in non‐lesional areas, which both associate with disease severity [15, 25]. The convergence of our findings with this expanding literature implicates vascular changes in disease pathogenesis beyond areas of demyelination in MS.

### 5.2 ECM protein accumulation in MS is modulated by HLA‐DRB1*15‐status

Decorin, biglycan and prolargin show a striking increase in parenchymal deposition in MS compared with controls. HLA‐DRB1*15‐positive MS cases had a significant increase in decorin and biglycan parenchymal expression compared to their HLA‐DRB1*15‐negative counterparts.

Our findings expand a rich literature demonstrating a role for ECM proteins in MS pathology, which has mostly focused on demyelinating lesions. Given the known pro‐inflammatory properties of these proteins, their increased parenchymal deposition in MS has important implications. In their soluble form, these proteins promote inflammation through Toll‐like receptor signalling, recruitment of pro‐inflammatory macrophages, and infiltration of T cells, which are thoroughly reviewed elsewhere [26], and are all well described features of MS pathology (Figure 1) [27, 28, 29]. No association was found in the current study between inflammatory markers of lymphocytic or microglia‐macrophage inflammation and parenchymal deposition of decorin or biglycan. However, pathology only provides a static view of a dynamic process. ECM remodelling may persist beyond the timeframe of transient inflammatory changes.

The provenance of parenchymal ECM deposition in MS is not clear. Could partial proteolytic processing of perivascular proteins and/or de novo synthesis by activated glial or immune cells in the parenchyma be the source? The striking reduction of decorin and biglycan perivascular expression with concomitant increased parenchymal expression in MS compared to controls would lend support to proteolytic processing of ECM proteins at the vascular interface. In support of this, the activation of matrix metalloproteinases secondary to leukocyte activation and extravasation due to blood–brain barrier disruption are known to cleave these proteins, which could explain the shift from perivascular to parenchymal expression in our MS cohort [16]. On the other hand, astrocytes, endothelial cells, leukocytes, and activated macrophages are known to produce soluble ECM proteins, which may be particularly relevant to prolargin where expression was largely restricted to MS cases in a vessel‐associated parenchymal pattern [16, 25, 26, 27, 28, 29, 30, 31]. Immune cell infiltration could explain the enhanced parenchymal expression of decorin and biglycan expression in spinal cord lesional areas [30]. Interestingly, the key regulator of ECM production, TGF‐beta, is bound in its latent form to fibrinogen, which could explain the altered vessel‐associated pattern described herein [32]. It is possible that the diffuse ECM remodelling observed in our study is a signature of cumulative past inflammatory activity in MS.

Another layer of complexity is the association of this parenchymal deposition with HLA status. ECM‐immune interactions may be exaggerated by genetic factors, particularly HLA‐DRB1*15 status. Several lines of evidence have shown more severe clinical, radiographic and pathological outcomes in MS harbouring the HLA‐DRB1*15 allele. The HLA‐DRB1*15 haplotype shapes the autoreactive T cell repertoire during thymic selection and increases autoreactive T‐cell proliferation [33, 34]. Incomplete deletion of autoreactive T cells or molecular mimicry with foreign antigens could be involved in the formation of an immune‐mediated response in MS modulated by HLA‐II class molecules. It is unlikely that ECM proteins act as auto‐antigens, given their widespread expression across different tissue types. Instead, the heightened predisposition to autoreactive inflammation of HLA‐DRB1*15‐positive leukocytes may have a synergistic effect with the known pro‐inflammatory profile of the identified ECM proteins and contribute to the pathological heterogeneity observed between people with MS. Notably, HLA‐DRB1*15 has been shown to interact with environmental risk factors such as smoking and obesity, influencing disease onset and severity. While these risk factors do not directly affect HLA class II molecules, they may create an inflammatory environment that amplifies the effect of HLA‐DRB1*15 by increasing antigen presentation [2]. Similarly, we can speculate that once proteolytically cleaved, ECM proteins might enhance inflammation and antigen presentation in the perivascular space and surrounding parenchyma. One must also consider the possibility that the ECM proteins were top proteomic hits with confirmed differences in their expression in histochemical analyses because of the higher inflammatory status associated with HLA‐DRB1*15 cases rather than a specific genotype effect. Future study exploring the interaction between genotype and these ECM proteins on pathological outcomes in MS warrants consideration.

### 5.3 Topographical expression of ECM proteins in controls may explain anatomical predilection of MS pathology

A curious feature of MS pathology is the predilection of demyelination and inflammation in certain sites throughout the CNS [10, 35, 36, 37, 38, 39, 40]. It is well described that the nature and extent of inflammation in lesional and non‐lesional areas differ between grey and white matter and across CNS sites. However, the cause of this CNS site variability is not known. Given the vasculocentric nature of MS pathology, it seems likely that factors in the vascular‐immunity axis underpin this variability. The striking difference in expression of these ECM proteins along the neuraxis in controls compared to MS provides an important clue. For example, in controls, prolargin and decorin expression are both essentially absent in the motor cortex but most abundant in the lumbar spinal cord. However, in MS, the motor cortex demonstrates heavy deposition of prolargin in the absence of any change in decorin expression while in the lumbar cord prolargin expression remains unchanged and decorin expression is markedly reduced. Non‐specific perivascular fibrotic change secondary to chronic inflammation cannot explain these findings.

Our findings fuel the hypothesis that topographic variation of MS pathology is modulated by antigen‐specific immune interactions with vessel‐associated ECM proteins. This premise is supported by other CNS inflammatory disorders where a relationship between antigen expression and disease topography have been demonstrated. For example, optic neuritis is common amongst people with neuromyelitis optica spectrum disorder. However, those with MOG‐antibodies have predominant anterior optic nerve involvement compared to those with AQP‐4‐antibodies who display more posterior optic chiasm and tract involvement [40, 41]. In MS, ECM proteins might prove an important contributor to the striking anatomical variation of its pathology.

### 5.4 Strengths and limitations

The current study has several strengths. Previous proteomic study in people with MS has implicated a growing number of protein candidates that could serve as potential disease‐specific markers amenable to targeted therapies [42, 43, 44, 45, 46, 47]. However, the biologic specificity and relevance of identified proteins to MS pathogenesis have been obscured by significant phenotypic (and likely pathologic) diversity in the clinical populations investigated. Additionally, where proteomic studies have used post‐mortem tissue in MS, the focus has been on methodology [46] or on one aspect of the proteome (e.g., mitochondrial proteome) [47] or on the analysis of tissue from demyelinating plaques [44]. We addressed these limitations by examining the proteome of well‐characterised post‐mortem MS cases with validation of top protein hits using objective and reproducible quantitative pathology methods. This provided a unique opportunity to visualise directly the distribution and extent of proteins selected a priori. The clinical phenotype of progressive MS is usually characterised by a motor syndrome predominantly affecting the lower limbs. The design of the study was to characterise blocks systematically along the motor pathway of the lower limbs. For this reason, the medial primary motor cortex (lower limb region), cervical and lumbar cord were studied. It is possible that other brain areas display a different pattern of expression of ECM proteins not explored herein. Finally, samples from other neuroinflammatory conditions were not included in this study due to tissue availability, which limits generalization of our findings and our ability to comment on whether these changes are specific to MS pathology.

We acknowledge limitations of the study of post‐mortem material. First, the discovery proteomic study was performed on a small sample size due the scarcity of suitable fresh frozen post‐mortem tissues and the decision to only include cases matched for demographic and pathological variables that could have biased the results. Second, we acknowledge the difficulty in quantifying total protein abundance in immunohistological analyses. This further justifies our approach of validating top proteomic hits using orthogonal methods, such as immunohistochemistry as undertaken herein.

In summary, we show striking differences in perivascular expression of three immunologically active ECM proteins, decorin, biglycan, and prolargin, throughout the length of the neuraxis, particularly in non‐lesional areas. These ECM proteins also accumulate in the parenchyma of MS cases, particularly those harbouring the HLA‐DRB1*15 allele. These findings point to ECM proteins and the vascular interface being key contributors to the landscape of MS pathology. Further, our observations provide a plausible link between genetic and immunopathogenic factors that are central to MS’ pathogenesis. In so doing, this study brings to light novel factors that may contribute to not only the pathogenesis of MS but also the heterogeneity of the topographical variation of MS pathology.

## AUTHOR CONTRIBUTIONS

Marco Pisa, Joseph L. Watson and Jonathan I. Spencer contributed to data acquisition, data analysis and manuscript writing. Guy Niblett, Yasamin Mahjoub, Andrew Lockhart, Richard L. Yates, Sydney A. Yee, Gina Hadley contributed to data acquisition and manuscript revision. Jennifer Ruiz, Margaret M. Esiri, Benedict Kessler, Roman Fischer and Gabriele C. DeLuca designed the study, supervised data acquisition and manuscript writing.

## FUNDING INFORMATION

The research was supported by the National Institute for Health Research (NIHR), Oxford Biomedical Research Centre (BRC), the Merck Serono Grant for MS Innovation, the Clarendon Fund and the Oxford‐Quinnipiac‐Trinity Health of New England partnership.

## CONFLICT OF INTEREST STATEMENT

The authors report no competing interests.

## Supporting information


**DATA S1.** Supporting Information.

## Data Availability

The data that support the findings of this study are available from the corresponding author, upon reasonable request.
