# Supplementary material for: A role for vessel‐associated extracellular matrix proteins in multiple sclerosis pathology
Source: Brain Pathol. 2024 Apr 25;34(6):e13263. doi: 10.1111/bpa.13263 (PMC11483522; doi:10.1111/bpa.13263)
Supplement: Supplementary file 1 — DATA S1. Supporting Information. [file BPA-34-e13263-s001.pdf]

Supplementary Materials

1. Supplementary methods

Supplementary 1A - Sample selection, preparation, and experimental procedure
Meningeal tissue was removed prior to tissue processing. Each spinal cord tissue specimen was washed in saline buffer to remove blood contaminants, ground into a powder with a mortar in Liquid Nitrogen, and homogenised using previously established methods.[1] Protein samples were extracted and digested with trypsin and subsequently desalted and subjected to state of the art nano liquid-chromatography tandem mass spectrometry (LC-MS/MS) (Orbitrap Fusion Lumos) using optimised methods and workflow parameters.[2] Mass spectrometry data sets were then processed, analysed, and quantified using Progenesis QI software (Waters). A merged peak-list generated by Progenesis LC-MS was searched against a merged Swissprot/Uniprot human database using the Mascot search engine (v. 2.5, http://www.matrixscience.com). Mascot based protein identifications were imported into Progenesis LC-MS using a peptide level false discovery rate of 1% and score cutoff of 20. Raw abundances were exported and used for Linear Models for Microarray (LIMMA) analysis in R. Raw protein abundance values from Progenesis QI were first normalised using quantile normalization, and technical replicates aggregated by taking the median normalised value for each protein in each sample. Differentially regulated proteins were used to build a logistic regression predictive model. Bootstrap forward-backward selection was performed using the boot.stepAOC R package to select proteins for inclusion in the final model. Statistical analysis of quantified proteins in the spinal cord tissue specimens from *HLA-DRB1*15* genotype groups were compared using Perseus and using R.[3] The analysis concentrated on identifying significant differences in protein expression between genotype groups at the spinal level sampled. Given the discovery design of the current study and the small sample size of the cohort, the selection of proteomic hits for the validation phase was based on multiple factors: the magnitude of change and the p-value of the difference between the *HLA-DRB1*15* subgroups, the presence of at least 2 unique peptides identified, and protein class clustering considerations.

Supplementary 1B – Formalin-fixed, paraffin-embedded tissue blocks were cut into 6-µm-thick adjacent sections for immunohistochemistry (IHC) using optimised method.[4] Briefly, adjacent sections were baked at 60°C for 20 minutes, deparaffinised in xylene and rehydrated with successive ethanol baths before removal of endogenous peroxidase. Antigen retrieval was done with microwave (two cycles of 5 minutes at maximal power) in a citrate pH 6 buffer. Adjacent sections were incubated with primary antibodies for: myelin (PLP, BioRad, # MCA839G), prolargin (PRELP, Abcam, #ab135595), decorin (DEC, Millipore, #MAB143), biglycan (BGN, Invitrogen, #PA5-82066). Subsequent labelling with secondary antibody and 3,3'-diaminobenzidine (DAB) visualization using the Dako Envision kit. The omission of primary and secondary antibodies separately were used as negative controls. Sections were counter-stained with haematoxylin.

	Antibody	Diluition	Incubation
PLP	BioRad, MCA839G	1:1000 (1µgr/mL) in TBS-T	1h room temperature
Decorin	Millipore, MAB143	1:100 (50µgr/mL) in TBS-T	1h room temperature
Biglycan	Invitrogen, PA5-82066	1:750 (7.5µgr/L) in 5% FCS	1h room temperature, after blocking with 10% FCS
Prolargin	Abcam, Ab135595	1:50 in TBS-T	1h room temperature
CD31/CD34	Dako, M0823 / BioRad, MCAP547	1:25 (40µg/mL) /1:50 (20µg/mL) in TBS-T	Overnight at 4'C

Supplementary 1C – Omission of the primary antibody was performed to confirm specificity of the immunohistochemistry analysis. The figure shows biglycan (B) and decorin (D) staining and the appearance of a consecutive section in case of omission of the primary antibody (A, C).

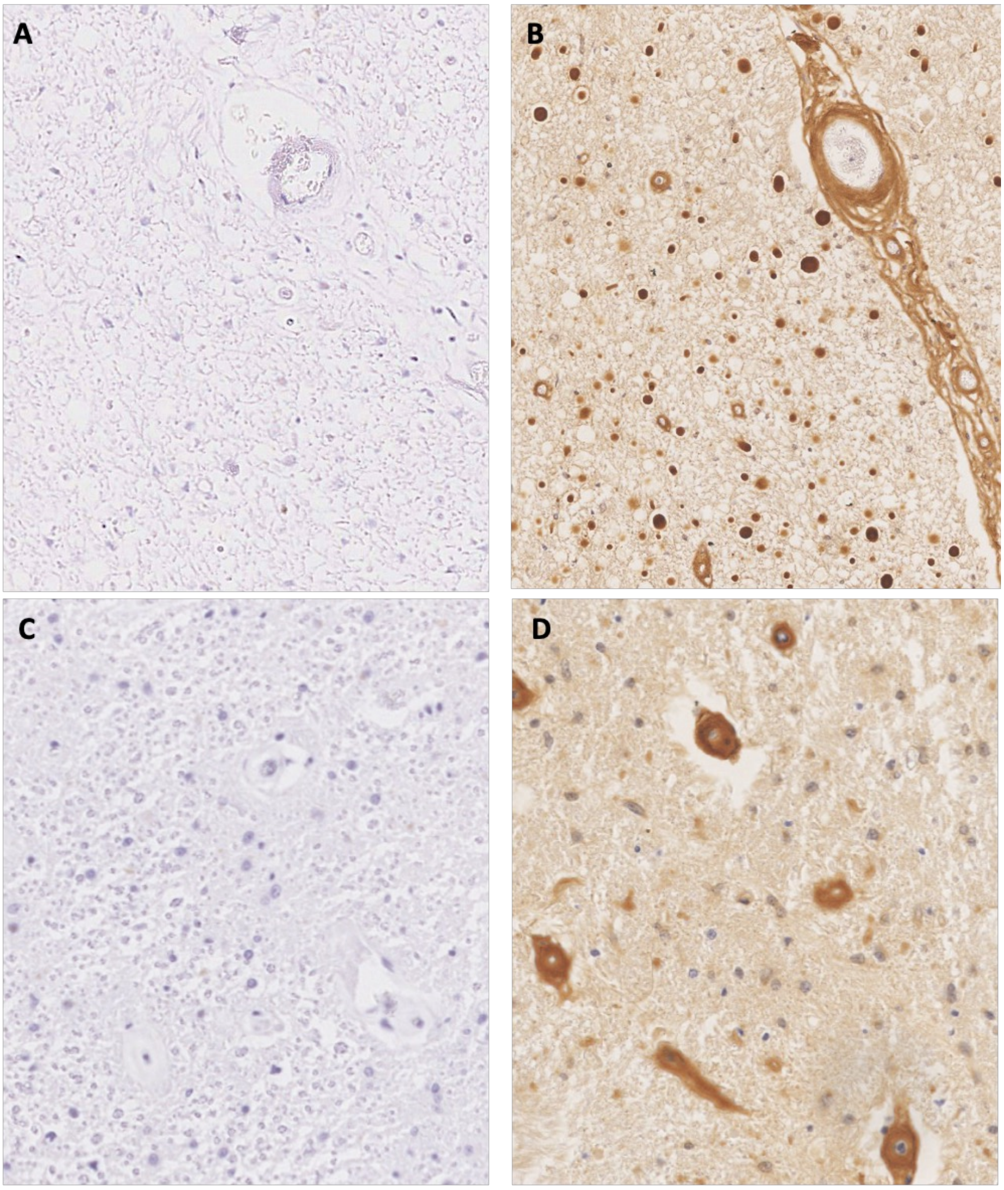

Supplementary 1D – All biglycan and decorin immunolabelled images were exported to Fiji.[5] RGB images and converted to 8-bit grayscale, appropriately thresholded (minimum intensity=163) and binarised, and perivascular biglycan or decorin was selectively detected and quantified using an optimised macro. The outer perimeter of vessels were detected using the Analyse Particles tool (size > 30 pixels^2, circularity > 0.2), and the lumen detected using the Analyse Particles tool on the inverse image (size > 5 pixels^2, circularity > 0.7). The ratio between the vessel (total-lumen) and lumenal area was then calculated to give the Area Index (AI) metric reported throughout the paper. This allowed systematic analysis of binarised vessels and their corresponding lumen, and the macro

parameters were chosen such that vessels were only analysed if cut in the transverse plane enclosing a single, unstained lumen.

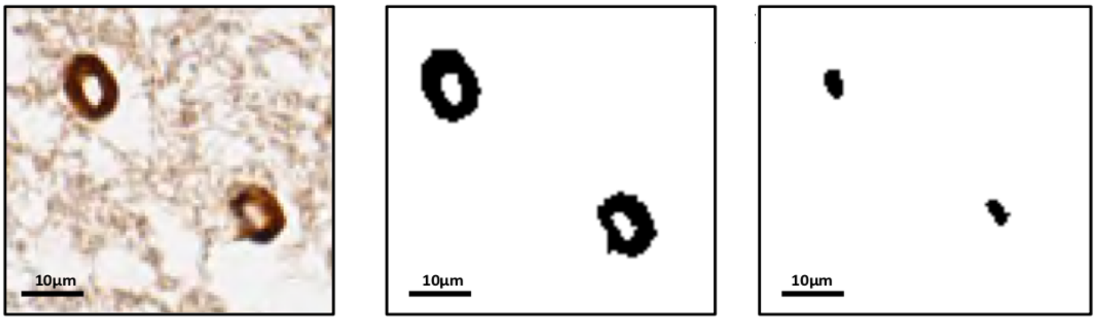

Supplementary 1E – Parenchymal staining was analysed semi-quantitatively by an assessor blinded to disease type and genetic status (JW), assigning each section a score of 0-2, independently validated by another blind observer (GD). This scoring scale was decided upon by the two observers (JW and GD) after blind assessment of a random subset of the images. Specifically, a score of 0 was determined to be nearly-completely free of parenchymal staining; a score of 2 was nearly total parenchymal staining, and a score of 1 was for intermediate cases. The two observers agreed that these three categories were the greatest resolution whereby cases could be quite unambiguously classified

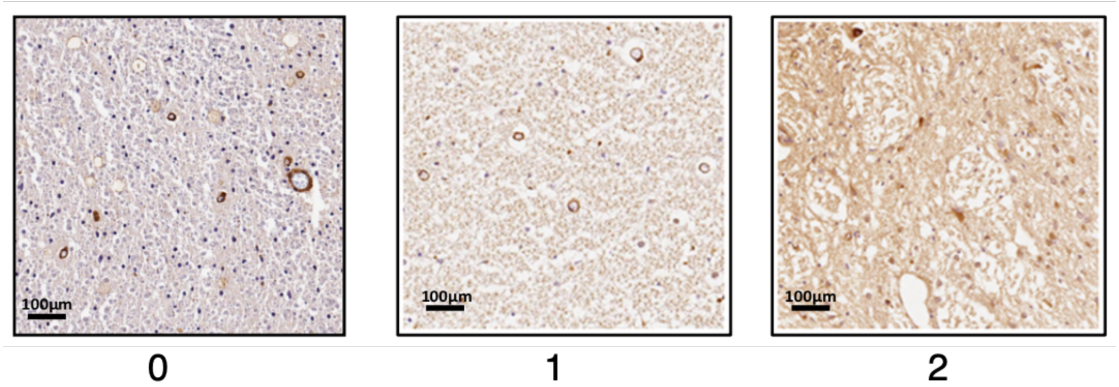

Supplementary 1F – Given the predominant perivascular expression of decorin and biglycan in the spinal cord, a systematic analysis of vascular coverage was undertaken. Sections were stained for CD31/CD34. Predetermined grey and white matter areas were imaged for each slide in ImageScope for export to Fiji. For each side, 5 snapshots taken of the lateral corticospinal tract, and 3 for each of the dorsal column and anterior horn. Each side (left or right) and area (lateral corticospinal tract, dorsal column or anterior horn) was determined to be lesional or normal appearing based on PLP-stained slides. All snapshots were then analysed using an ImageJ macro. Images were converted to 8-bit, thresholded based on pixel intensity, then proportional vessel area and number was counted using the Analyze Particles tool based on thresholded CD31/34-stained vessels. Total area of measured particles per ImageScope snapshot was used as a metric of vascular coverage, thus accounting for both size and number of vessels. Means of vascular area for each cases' spinal cord level and matter-type were then determined.

Double-labelled immunohistochemistry was used to assess whether perivascular changes in ECM protein expression was affecting arterioles, venules or both. Smooth muscle actin was used to visualize arterioles. Given that decorin and biglycan showed co-localisation around vessels, co-expression of biglycan (ThermoFisher, #PA5-82066) and prolargin (Abcam,

#ab135595) with smooth muscle actin (Dako, #M0851) were evaluated by double-labelled immunohistochemistry using AlexaFluor secondary antibodies (i.e. Alexa Fluor 594 (anti-mouse, ThermoFisher, #A-21203) and Alexa Fluor 647 (anti-rabbit, ThermoFisher, #A32795), respectively) imaged by an Olympus FV1000 confocal microscope at 20X magnification (Supplementary 3F and 5E).

Supplementary 1G – Statistical analyses were performed using SPSS version 26 (SPSS, Chicago, IL). Graphs were drawn using GraphPad Prism version 9.2 (GraphPad Software, San Diego, California, USA), with all bars illustrated as mean±SEM. As described, parenchymal distribution of biglycan and decorin was assessed using an ordinal semi-quantitative score. The area index used as a measure of perivascular biglycan and decorin was a proportional metric, and prolargin expression was quantified as a positive pixel count. Vascular area was a proportional average area of slide snapshots. We assessed differences in the distribution of these variables across CNS areas and between below specified subgroups of cases using Generalized Estimating Equation (GEE) models taking into account random subject-related variability.

Generalised Estimating Equation linear models were built considering biglycan area index, decorin area index and prolargin pixel count as dependent variables. Disease status (MS vs controls), HLA status (*HLA-DRB1*15* positive vs negative), Site (cortex, lumbar and cervical spinal cord area), Matter type (gray vs white matter) and Lesion status (lesional vs normal-appearing) were considered in the models as independent factors together with their interaction terms. Possible factorial analyses were limited to those selected in a predefined pipeline. In controls, we assessed the effects of site, matter type, and their interaction. In the overall autopsy cohort (MS plus controls) we assessed the effects of disease status, site, and their interaction with matter type and Lesion status. In the MS cohort, we assessed the effect of HLA status and its interaction with site and matter type. Sensitivity analyses were run including vascular area as a covariate in Generalized Estimating Equation models.

We report the contribution of each independent factor or interaction term to the model with the Wald Chi-square and its p-value. Pairwise comparison of estimated means between factor or interaction terms is reported as a p-value, adjusted for multiple comparisons using a least significant difference (LSD) correction. To give an estimate of the magnitude of change between subgroups, we report the percentage change (Difference between estimated means of the two groups / Estimated mean of reference group). The value of the estimated means of each dependent variable together with their 95% Wald confidence interval is reported in supplementary materials as indicated.

As the parenchymal distribution of biglycan and decorin was expressed using an ordinal score ranging from 0 to 2, its association with Disease status (MS vs controls), HLA status (*HLA-DRB1*15* positive vs negative), and site (cortex, lumbar and cervical spinal cord area) was assessed using cross-tab analyses. Given the ordinal distribution of each term, a symmetric Somer's D test was chosen and its approximate significance p-value is reported in the text. For all analyses, statistical significance is set at $P<0.05$.

2. Shotgun Proteomics

Supplementary 2A – Characteristics of the cases used for the discovery proteomic analysis of fresh frozen cervical cord tissue. Cases were selected based on tissue availability and were matched for global spinal cord plaque area and clinico-demographic characteristics. All the five fresh frozen blocks sampled showed mixed active-inactive lesions; average total microglia-macrophage infiltration (CD68+ area coverage on total tissue area, %) and proportional demyelinating area (%) did not differ between genotype groups.

	HLA-DRB1*15-positive (n=3)	HLA-DRB1*15-negative (n=2)
Age (years)	58.67	44.50
Sex	2M:1F	1M:1F
Disease Duration (years)	26.67	22.00
MS type	3 SPMS	2 SPMS
Brain weight (gr)	1231.33	1177.00
Post-mortem interval (hours)	24.00	19.50
Global plaque area (%)	36.61	31.83
Cervical plaque area (%)	46.41%	30.77%
Cervical CD68+ area coverage (%)	1.95%	2.47%

Supplementary 2B – Discovery shotgun proteomics on fresh frozen cervical spinal cord from HLA-DRB1*15-positive (n=3) and -negative (n=2) MS cases matched for lesion load identified 1,656 protein groups of which 804 were quantified with two peptides or more with 99 identified proteins differing between genotype groups (P<0.01). The top 3 differentially-expressed proteins between HLA-DRB1*15 subgroups were small-leucine rich proteoglycan ECM proteins, namely decorin, biglycan and prolargin, which were all over-expressed in HLA-DRB1*15-positive cases. Three extracellular matrix proteins, namely biglycan, prolargin and decorin were the top differentially expressed proteins between HLA-DRB1*15-positive versus -negative MS cases. (A) Volcano plot demonstrating upregulated protein expression in HLA-DRB1*15-positive versus negative cases: biglycan (14 peptides, 8.5-fold), prolargin (8 peptides, 7.6-fold) and decorin (5 peptides, 4.2-fold). (B) Euclidian clustering of quantified proteins. Cluster analysis separates HLA-DRB1*15-positive versus negative MS cases. Biglycan, decorin and prolargin are consistently up-regulated in HLA-DRB1*15- positive cases across all cases. (C) Pearson correlation shows good technical agreement between the spinal cord proteomes of each MS case studied (n=5). (D) The table reports the top nine protein hits together with the log^2 fold change and -log10 p-value of the difference between HLA-DRB1*15 subtypes; the number of unique peptides identified and the median peak intensity within each sample are also reported.

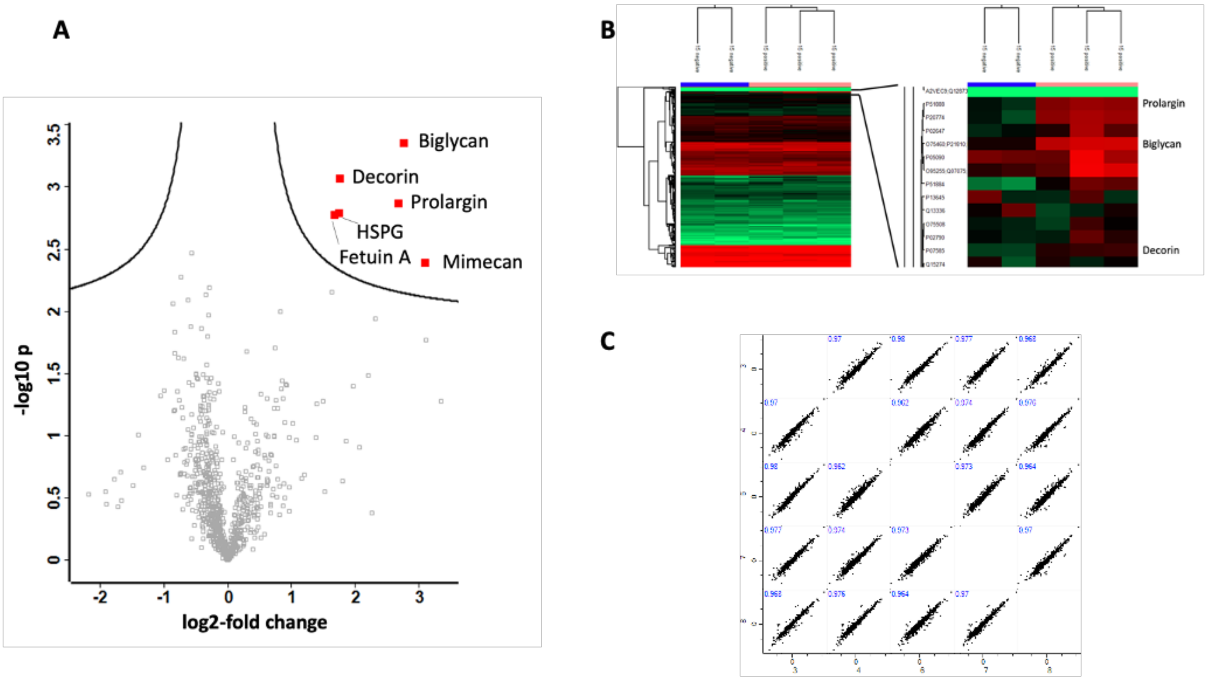

D				Log transformed and median substracted intensities of summed duplicate measurements						
				HLA-DR15-positive			HLA-DR15-negative			
Significant	-LOG (P-value)	Difference (log2 fold change)	Unique peptides	Sample 3	Sample 6	Sample 7	Sample 4	Sample 8	Gene name	Leading protein name
+	2.78	1.68	2	-1.206301	-1.174446	-1.438038	-3.099566	-2.801024	AHSG FETUA PRO2743	Fetuin-A
+	2.79	1.75	2	-1.954941	-1.91996	-2.170096	-3.933149	-3.601378	HSPG2	HSPG
+	3.07	1.76	5	0.9709988	0.8837166	0.6402988	-0.9579124	-0.9060688	DCN SLRR1B	Decorin
+	2.87	2.68	8	2.251608	2.397884	2.028896	-0.7032299	-0.2108383	PRELP SLRR2A	Prolargin
+	2.39	3.10	8	2.423464	2.660326	2.176102	-1.140558	-0.2215977	OGN OIF SLRR3A	Mimecan
+	3.36	2.77	14	3.286142	3.311209	2.921221	0.3958645	0.4074459	BGN	Biglycan
	1.77	3.12	5	1.497496	1.775879	0.2081261	-2.189	-1.733419	LUM LDC SLRR2D	Lumican
	2.15	1.64	4	-0.8723984	-1.081848	-0.4318275	-2.423887	-2.438425	FMOD FM SLRR2E	Fibromodulin
	1.94	2.32	2	-3.183688	-3.716789	-3.742329	-5.409255	-6.332208	COL14A1 UND	Collagen alpha-1

3. Cohort characteristics and pathological outcomes

Supplementary Table 3A – Proportion of samples displaying white matter (WM) and gray matter lesions (GM) and total proportional demyelinated area are reported according to *HLADRB1*15* status. Lesion staging is reported according to prior guidelines.[6]

% samples	HLA-DRB1*15-negative N = 45	HLA-DRB1*15-positive N = 32
WM Acute	7.5%	8.2%
WM Mixed active-inactive	23.9%	28.6%
WM Inactive	11.9%	8.2%
WM total active lesion	25.4%	28.6%
WM total lesions	29.9%	34.7%
GM Acute	6.0%	4.1%
GM Inactive	17.9%	16.3%
GM Mixed active-inactive	19.4%	24.5%
GM total active lesion	23.9%	24.5%
GM total lesions	29.9%	30.6%
Proportional demyelinated area	12.78% (CI95% 4.22 - 21.33)	21.7% (CI95% 9.13 - 34.27%)

Supplementary Table 3B – Pathological outcomes of inflammation in the non-lesional white matter (NLWM) and non-lesional gray matter (NLGM) are reported according to *HLADRB1*15* status. Lymphocytic CD3+, CD8+ and CD20+ inflammation was scored as number of positive cells per 400X field of view in predefined region of interest across the NLWM and NWGM, as described elsewhere.[7] CD68+ microglia-macrophage infiltration was quantified in the same regions of interest using a positive pixel count macro.

	*HLADRB1*15-negative*		*HLADRB1*15-positive*	
	Mean	*95% Wald Confidence Interval*	*Mean*	*95% Wald Confidence Interval*
CD3+ NLWM	4.57	*(2.43 - 6.70)*	5.31	*(3.45 - 7.18)*
CD3+ NLGM	3.86	*(2.05 - 5.66)*	4.65	*(2.61 - 6.69)*
CD8+ NLWM	3.77	*(2.63 - 4.91)*	4.19	*(2.91 - 5.46)*
CD8+ NLGM	1.71	*(0.93 - 2.48)*	2.32	*(0.94 - 3.69)*
CD20+ NLWM	0.05	*(0.00 - 0.10)*	0.12	*(0.02 - 0.23)*
CD20+ NLGM	0.00	*(0.00 - 0.00)*	0.06	*(-0.02 - 0.14)*
CD68+ NLWM	69837.20	*(50652.14 - 89022.26)*	65676.16	*(48380.97 - 82971.36)*
CD68+ NLGM	66311.78	*(48508.63 - 84114.93)*	56837.86	*(45115.61 - 68560.11)*

4. Vascular area

Supplementary Table 4A – Total vascular area in non-neurological controls according to matter type (GM, gray matter; WM, white matter) and site (primary motor cortex, cervical and lumbar cord).

				95% Wald Confidence Interval	
Matter type	Site	Mean	Std. Error	Lower	Upper
GM	Cervical	21636.4444	1421.01111	18851.3139	24421.5750
	Cortex	46484.0694	5040.15358	36605.5500	56362.5889
	Lumbar	23220.6667	2045.21375	19212.1214	27229.2120
WM	Cervical	7187.03333	975.341421	5275.39928	9098.66739
	Cortex	21363.1111	4333.57392	12869.4623	29856.7599
	Lumbar	5937.43889	617.539905	4727.08292	7147.79486

Supplementary Table 4B – Total vascular area in MS and non-neurological controls according to according to matter type (GM, gray matter; WM, white matter), site (primary motor cortex, cervical and lumbar cord), and tissue type (non-neurological control tissue, lesional multiple sclerosis tissue, and normal appearing multiple sclerosis tissue).

Matter type	Site	Area type	Mean	Std. Error	95% Wald Confidence Interval	
					Lower	Upper
GM	Cervical	Controls	21636.4444	1421.01111	18851.3139	24421.5750
		MS, Lesional area	13453.3590	1016.34961	11461.3503	15445.3676
		MS, Normal appearing area	13899.3205	851.079685	12231.2350	15567.4060
	Cortex	Controls	46484.0694	5040.15358	36605.5500	56362.5889
		MS, Lesional area	30651.1675	2950.46343	24868.3654	36433.9696
		MS, Normal appearing area	33887.6020	2442.62045	29100.1539	38675.0502
	Lumbar	Controls	23220.6667	2045.21375	19212.1214	27229.2120
		MS, Lesional area	17838.6364	1391.88835	15110.5853	20566.6874
		MS, Normal appearing area	17679.9023	857.130494	15999.9574	19359.8472
WM	Cervical	Controls	7187.03333	975.341421	5275.39928	9098.66739
		MS, Lesional area	8679.64497	1017.32248	6685.72955	10673.5604
		MS, Normal appearing area	8061.09167	914.306140	6269.08456	9853.09877
	Cortex	Controls	21363.1111	4333.57392	12869.4623	29856.7599
		MS, Lesional area	20254.8137	1619.77642	17080.1103	23429.5172
		MS, Normal appearing area	23250.8042	1577.95046	20158.0781	26343.5302
	Lumbar	Controls	5937.43889	617.539905	4727.08292	7147.79486
		MS, Lesional area	8098.16563	1144.30801	5855.36314	10340.9681
		MS, Normal appearing area	7052.22241	518.226162	6036.51779	8067.92702

Supplementary Table 4C – Total vascular area in multiple sclerosis cases according to *HLA-DRB1*15* status and matter type (GM, gray matter; WM, white matter).

Matter type	HLA status	Mean	Std. Error	95% Wald Confidence Interval	
				Lower	Upper
GM	MS, HLA-DRB1*15 positive	23423.5803	1811.34084	19873.4175	26973.7431
	MS, HLA-DRB1*15 negative	20898.4725	1551.23488	17858.1080	23938.8370
WM	MS, HLA-DRB1*15 positive	14247.7877	1405.25697	11493.5347	17002.0407
	MS, HLA-DRB1*15 negative	11269.0318	814.689833	9672.26908	12865.7945

5. Biglycan

Supplementary Figure 5A – A variable degree of glial expression of biglycan was seen, predominantly in the subpial layers, in both motor cortex and spinal cord in multiple sclerosis and controls. The panel on the left shows a cluster of perivascular glial cells with cytoplasmic biglycan; the panel on the right shows sparse star-shaped glial cells in the subpial white matter (pictures taken at 40X magnification).

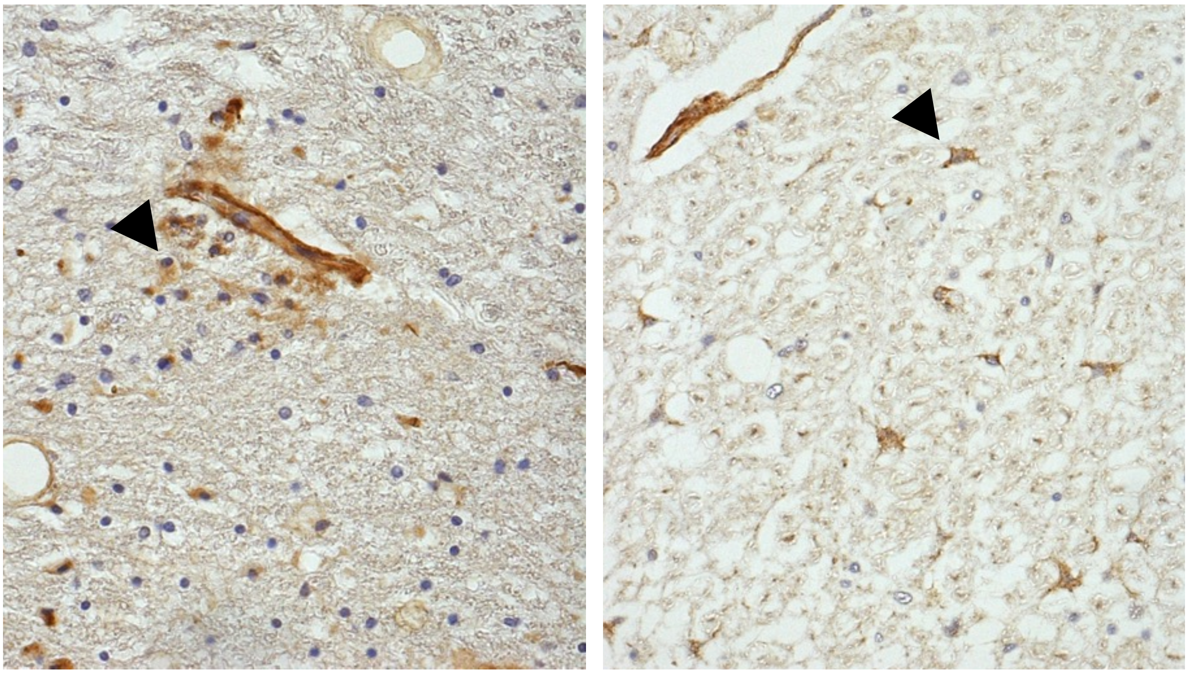

Supplementary Table 5B – Perivascular biglycan area index values in non-neurological controls according to matter type (GM, gray matter; WM, white matter) and site (primary motor cortex, cervical and lumbar cord).

Matter type	Site	Mean	Std. Error	95% Wald Confidence Interval	
				Lower	Upper
GM	Cervical	8.51042747	.992336489	6.56548369	10.4553712
	Lumbar	9.34344586	2.15475327	5.12020706	13.5666847
WM	Cervical	12.7720512	2.13919737	8.57930139	16.9648010
	Lumbar	10.8895321	2.37226112	6.23998573	15.5390784

Supplementary Table 5C – Perivascular biglycan immunoreactivity area index values in MS and non-neurological controls according to according to matter type (GM, gray matter; WM, white matter), site (cervical and lumbar cord), and tissue type (non-neurological control tissue, lesional multiple sclerosis tissue, and normal appearing multiple sclerosis tissue).

Matter type	Site	Area type	Mean	Std. Error	95% Wald Confidence Interval	
					Lower	Upper
GM	Cervical	Controls	8.51042747	.992336489	6.56548369	10.4553712
		MS, Lesional area	6.05639204	.523960016	5.02944927	7.08333480
		MS, Normal appearing area	5.37777358	.245299713	4.89699498	5.85855218
	Lumbar	Controls	9.34344586	2.15475327	5.12020706	13.5666847
		MS, Lesional area	6.68130766	1.17594079	4.37650607	8.98610925
		MS, Normal appearing area	6.69700683	.351960408	6.00717711	7.38683655
WM	Cervical	Controls	12.7720512	2.13919737	8.57930139	16.9648010
		MS, Lesional area	7.59585639	.803685461	6.02066183	9.17105095
		MS, Normal appearing area	6.52628114	.466465119	5.61202630	7.44053597
	Lumbar	Controls	10.8895321	2.37226112	6.23998573	15.5390784
		MS, Lesional area	7.30598750	1.91145984	3.55959507	11.0523799
		MS, Normal appearing area	7.31972798	.587856426	6.16755056	8.47190540

Supplementary Table 5D – Perivascular biglycan immunoreactivity area index values in MS cases according to *HLA-DRB1*15* status, and site (cervical and lumbar cord).

Site	HLA status	Mean	Std. Error	95% Wald Confidence Interval	
				Lower	Upper
Cervical	MS, HLA-DRB1*15 positive	6.55426374	.527086220	5.52119374	7.58733375
	MS, HLA-DRB1*15 negative	6.07040696	.520403665	5.05043452	7.09037940
Lumbar	MS, HLA-DRB1*15 positive	6.84887799	.798339185	5.28416194	8.41359404
	MS, HLA-DRB1*15 negative	7.17054403	.584392614	6.02515555	8.31593250

Supplementary Table 5E – Perivascular biglycan immunoreactivity area index values in demyelinated areas according to *HLA-DRB1*15* status.

HLA status	Mean	Std. Error	95% Wald Confidence Interval	
			Lower	Upper
MS, HLADRB1*15-negative	7.31880274	0.94161780	5.47326577	9.16433972
MS, HLADRB1*15-positive	5.78218399	0.34235334	5.11118378	6.45318420

Supplementary Table 5F – Perivascular biglycan immunoreactivity area index values in demyelinated areas according to plaque stage (active, n=2; mixed active-inactive, n=22; inactive, n= 8; multiple plaques within same block, n=15).

Plaque stage	Mean	Std. Error	95% Wald Confidence Interval	
			Lower	Upper
Active	5.22617056	0.00000003	5.22617050	5.22617062
Inactive	7.69918572	1.61315358	4.53746280	10.8609086
Mixed	7.63771160	0.74898052	6.16973675	9.10568645
Multiple	5.63890559	0.86784423	3.93796215	7.33984902

Supplementary Table 5G – Distribution of parenchymal biglycan semiquantitative score in controls and MS cases divided according to *HLA-DRB1*15* status.

			Parenchymal Biglycan Score			Total
			0	1	2	
HLADRB15_Status	MS, HLA-DRB1*15 positive	Count	4	15	12	31
		% within HLADRB15_Status	12.9%	48.4%	38.7%	100.0%
	MS, HLA-DRB1*15 negative	Count	17	16	9	42
		% within HLADRB15_Status	40.5%	38.1%	21.4%	100.0%
	Controls	Count	6	6	0	12
		% within HLADRB15_Status	50.0%	50.0%	0.0%	100.0%
Total		Count	27	37	21	85
		% within HLADRB15_Status	31.8%	43.5%	24.7%	100.0%

Supplementary Figure 5H – Biglycan is expressed in the Virchow Robin spaces of both arterioles and venules. The picture shows double labelling of smooth muscle actin (Dako, #M0851) and biglycan (ThermoFisher, #PA5-82066) in a spinal cord sample from an MS case. Similar findings were observed throughout the neuraxis in both MS and control cases. Double-labelling was performed using AlexaFluor secondary antibodies (i.e. Alexa Fluor 594

(anti-mouse, ThermoFisher, #A-21203) and Alexa Fluor 647 (anti-rabbit, ThermoFisher, #A32795), respectively) imaged by an Olympus FV1000 confocal microscope at 20X magnification.

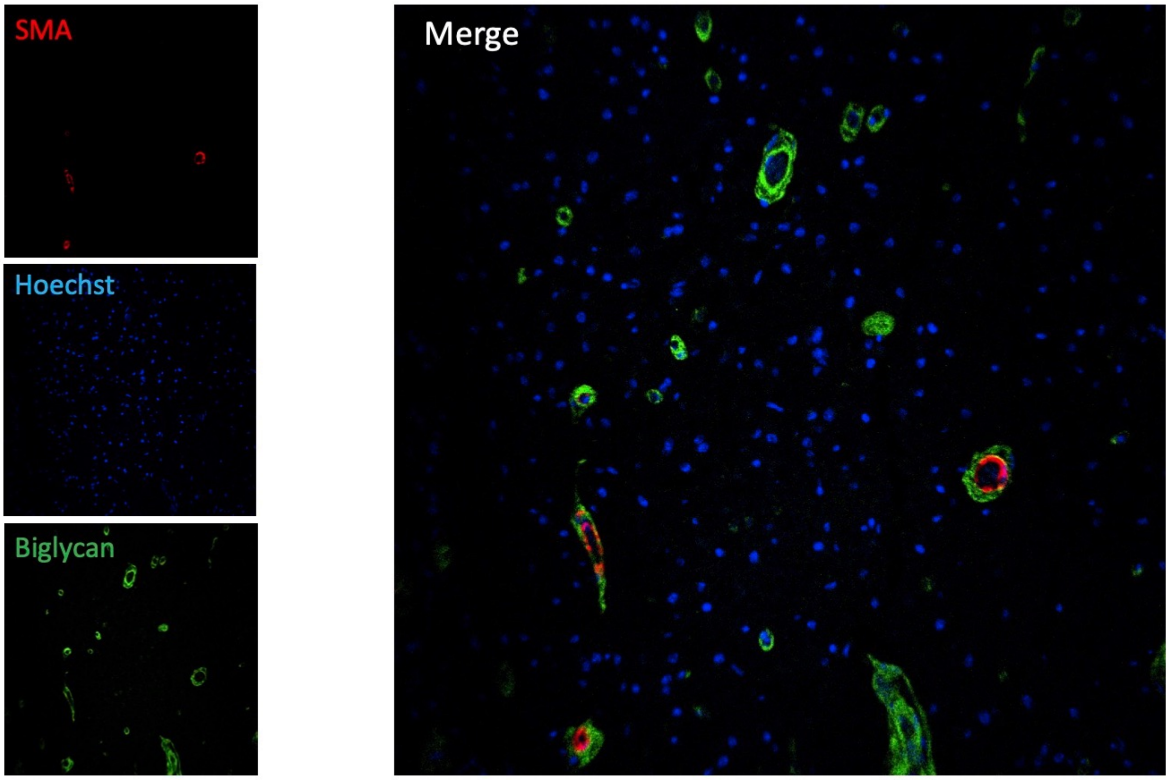

6. Decorin

Supplementary Table 6A – Perivascular decorin area index values in non-neurological controls according to matter type (GM, gray matter; WM, white matter) and site (primary motor cortex, cervical and lumbar cord).

Matter type	Site	Mean	Std. Error	95% Wald Confidence Interval	
				Lower	Upper
GM	Cervical	6.65364751	.575156084	5.52636230	7.78093272
	Lumbar	11.0655631	1.59137779	7.94651994	14.1846063
WM	Cervical	9.91987916	1.62962923	6.72586456	13.1138938
	Lumbar	12.9908086	1.59577718	9.86314284	16.1184744

Supplementary Table 6B – Perivascular decorin immunoreactivity area index values in MS and non-neurological controls according to according to matter type (GM, gray matter; WM, white matter), site (cervical and lumbar cord), and tissue type (non-neurological control tissue, lesional multiple sclerosis tissue, and normal appearing multiple sclerosis tissue).

Matter type	Site	Area type	Mean	Std. Error	95% Wald Confidence Interval	
					Lower	Upper
GM	Cervical	Controls	6.65364751	.575156084	5.52636230	7.78093272
		MS, Lesional area	7.90575727	.807454366	6.32317579	9.48833874
		MS, Normal appearing area	6.02991713	.280169311	5.48079537	6.57903889
	Lumbar	Controls	11.0655631	1.59137779	7.94651994	14.1846063
		MS, Lesional area	7.80853553	.602820455	6.62702915	8.99004192
		MS, Normal appearing area	5.75792618	.453475901	4.86912975	6.64672262
WM	Cervical	Controls	9.91987916	1.62962923	6.72586456	13.1138938
		MS, Lesional area	9.77914383	1.14829424	7.52852847	12.0297592
		MS, Normal appearing area	9.10695382	.660172011	7.81304046	10.4008672
	Lumbar	Controls	12.9908086	1.59577718	9.86314284	16.1184744
		MS, Lesional area	8.95652549	.969184066	7.05695962	10.8560914
		MS, Normal appearing area	7.89496179	.451747632	7.00955270	8.78037088

Supplementary Table 6C – Perivascular decorin immunoreactivity area index values in MS cases according to *HLA-DRB1*15* status, and site (cervical and lumbar cord).

Site	HLA status	Mean	Std. Error	95% Wald Confidence Interval	
				Lower	Upper
Cervical	MS, HLA-DRB1*15 positive	8.60631144	.574832620	7.47966021	9.73296267
	MS, HLA-DRB1*15 negative	7.66639117	.688575827	6.31680735	9.01597499
Lumbar	MS, HLA-DRB1*15 positive	7.36758140	.588695230	6.21375995	8.52140285
	MS, HLA-DRB1*15 negative	7.20641381	.518741340	6.18969946	8.22312815

Supplementary Table 6D – Perivascular decorin immunoreactivity area index values in demyelinated areas according to *HLA-DRB1*15* status.

HLA status	Mean	Std. Error	95% Wald Confidence Interval	
			Lower	Upper
MS, HLADRB1*15-negative	8.17762714	0.42384603	7.34690418	9.00835011
MS, HLADRB1*15-positive	7.54775896	0.74726316	6.08315008	9.01236784

Supplementary Table 6E – Perivascular decorin immunoreactivity area index values in demyelinated areas according to plaque stage (active, n=2; mixed active-inactive, n=25; inactive, n= 8; multiple plaques within same block, n=18).

Plaque stage	Mean	Std. Error	95% Wald Confidence Interval	
			Lower	Upper
Active	5.99429738	0.62136044	4.77645330	7.21214146
Inactive	6.70932375	0.80713874	5.12736089	8.29128661
Mixed	10.2860509	1.21059465	7.91332901	12.6587728
Multiple	8.46110017	0.53351027	7.41543925	9.50676108

Supplementary Table 6F – Distribution of parenchymal decorin semiquantitative score in controls and MS cases divided according to *HLA-DRB1*15* status.

		Parenchymal Decorin Score			Total
		0	1	2	
MS, HLA-DRB1*15 positive	Count	8	17	5	30
	% within HLADRB15_Status	26.7%	56.7%	16.7%	100.0%
MS, HLA-DRB1*15 negative	Count	22	16	5	43
	% within HLADRB15_Status	51.2%	37.2%	11.6%	100.0%
Controls	Count	10	1	0	11
	% within HLADRB15_Status	90.9%	9.1%	0.0%	100.0%
Total	Count	40	34	10	84
	% within HLADRB15_Status	47.6%	40.5%	11.9%	100.0%

7. Prolargin

Supplementary Figure 7A – Multiple sclerosis cases consistently showed striking extracellular perivascular prolargin immunoreactivity, as seen in the figure on the left. A granular staining pattern was also observed in the parenchyma in the neuronal and glial cytoplasm. Black arrows and dashed line inset indicate glial cells, while the arrowheads and continuous line inset indicate neurons (left figure, 20X magnification; right figure 40X magnification).

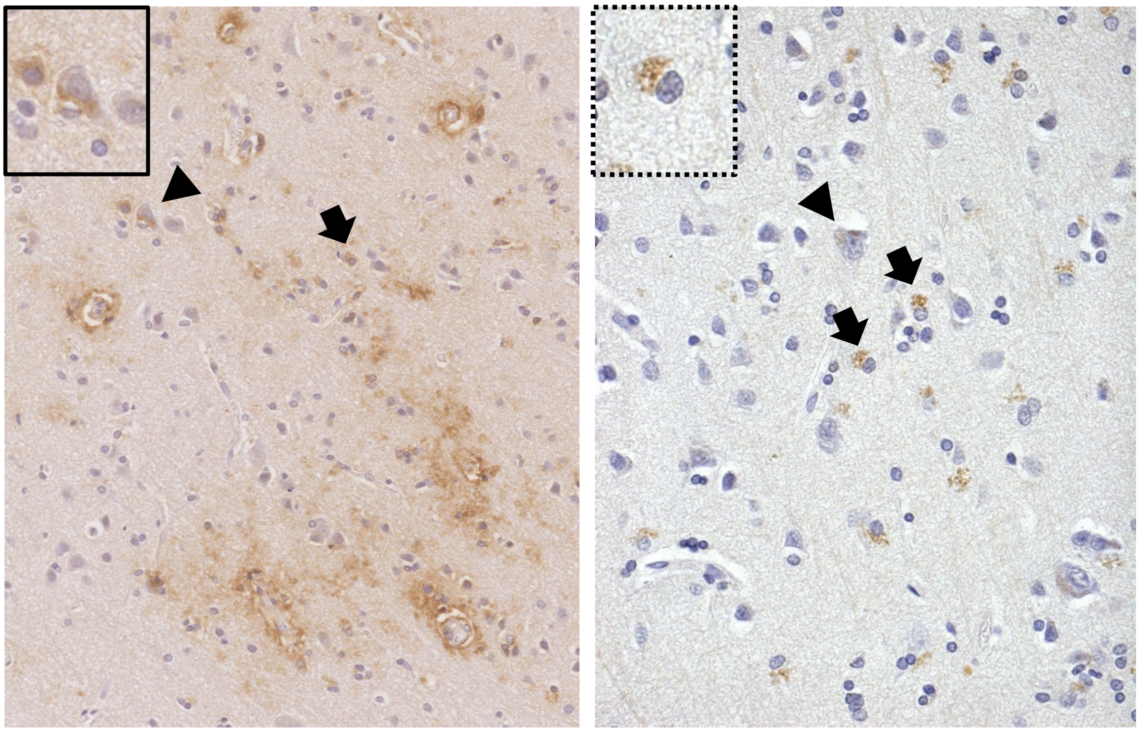

Supplementary Table 7B – Prolargin positive pixel counts in non-neurological controls according to matter type (GM, gray matter; WM, white matter) and site (primary motor cortex, cervical and lumbar cord).

Matter type	Site	Mean	Std. Error	95% Wald Confidence Interval	
				Lower	Upper
GM	Cervical	.096661847	.010802767	.075488814	.117834880
	Cortex	.039263053	.004449406	.030542379	.047983728
	Lumbar	.138758084	.009626649	.119890199	.157625968
WM	Cervical	.118444139	.009747567	.099339259	.137549019
	Cortex	.041008757	.009943724	.021519416	.060498098
	Lumbar	.182424187	.024537211	.134332136	.230516237

Supplementary Table 7C – Prolargin positive pixel counts in MS and non-neurological controls according to according to matter type (GM, gray matter; WM, white matter), site (cervical and lumbar cord), and tissue type (non-neurological control tissue, lesional multiple sclerosis tissue, and normal appearing multiple sclerosis tissue).

Matter type	Site	Area type	Mean	Std. Error	95% Wald Confidence Interval	
					Lower	Upper
GM	Cervical	Controls	.096661847	.010802767	.075488814	.117834880
		MS, Lesional area	.153793919	.036097314	.083044483	.224543355
		MS, Normal appearing area	.144382221	.022344883	.100587055	.188177387
	Cortex	Controls	.039263054	.004449406	.030542379	.047983728
		MS, Lesional area	.075816633	.010325181	.055579649	.096053617
		MS, Normal appearing area	.068528085	.010547079	.047856191	.089199979
	Lumbar	Controls	.138758084	.009626649	.119890199	.157625968
		MS, Lesional area	.088627797	.014091537	.061008892	.116246701
		MS, Normal appearing area	.125843967	.016506968	.093490906	.158197029
WM	Cervical	Controls	.118444139	.009747567	.099339259	.137549019
		MS, Lesional area	.106454545	.017646918	.071867220	.141041869
		MS, Normal appearing area	.216192271	.021290538	.174463585	.257920958
	Cortex	Controls	.041008757	.009943724	.021519416	.060498098
		MS, Normal appearing area	.091686864	.011727736	.068700923	.114672804
	Lumbar	Controls	.182424187	.024537211	.134332136	.230516237
		MS, Lesional area	.103701785	.020279974	.063953767	.143449803
		MS, Normal appearing area	.181874545	.013961485	.154510538	.209238553

Supplementary Table 7D – Prolargin positive pixel count in MS cases according to *HLA-DRB1*15* status, and site (cervical and lumbar cord).

Site	HLA status	Mean	Std. Error	95% Wald Confidence Interval	
				Lower	Upper
Cortex	MS, HLA-DRB1*15 positive	.078154900	.013734762	.051235261	.105074539
	MS, HLA-DRB1*15 negative	.079240087	.013222175	.053325101	.105155074
Cervical	MS, HLA-DRB1*15 positive	.159371361	.029642428	.101273271	.217469452
	MS, HLA-DRB1*15 negative	.161823670	.026603554	.109681663	.213965678
Lumbar	MS, HLA-DRB1*15 positive	.145583003	.018230152	.109852562	.181313445
	MS, HLA-DRB1*15 negative	.134240101	.018374319	.098227097	.170253105

Supplementary Table 7D – Prolargin positive pixel count in demyelinated areas according to *HLA-DRB1*15* status.

HLA status	Mean	Std. Error	95% Wald Confidence Interval Lower	Upper
MS, HLADRB1*15-negative	0.10507589	0.01339153	0.07882898	0.13132280
MS, HLADRB1*15-positive	0.09969001	0.01219821	0.07578196	0.12359805

Supplementary Table 6E – Prolargin positive pixel count in demyelinated areas according to plaque stage (active, n=4; mixed active-inactive, n=27; inactive, n= 6; multiple plaques within same block, n=20).

Plaque stage	Mean	Std. Error	95% Wald Confidence Interval Lower	Upper
Active	0.10572335	0.00306960	0.09970705	0.11173965
Inactive	0.09748446	0.01868964	0.06085343	0.13411549
Mixed	0.12645000	0.02555900	0.07635527	0.17654473
Multiple	0.09357618	0.01646410	0.06130715	0.12584522

Supplementary Figure 7E – Prolargin deposition was observed outside the Virchow Robin spaces of both arterioles and venules in the motor cortex in MS cases. The picture shows double labelling of smooth muscle actin (Dako, #M0851) and prolargin (Abcam, #ab135595) in a motor cortex sample from an MS case. Double-labelling was performed using AlexaFluor secondary antibodies (i.e. Alexa Fluor 594 (anti-mouse, ThermoFisher, #A-21203) and Alexa Fluor 647 (anti-rabbit, ThermoFisher, #A32795), respectively) imaged by an Olympus FV1000 confocal microscope at 20X magnification.

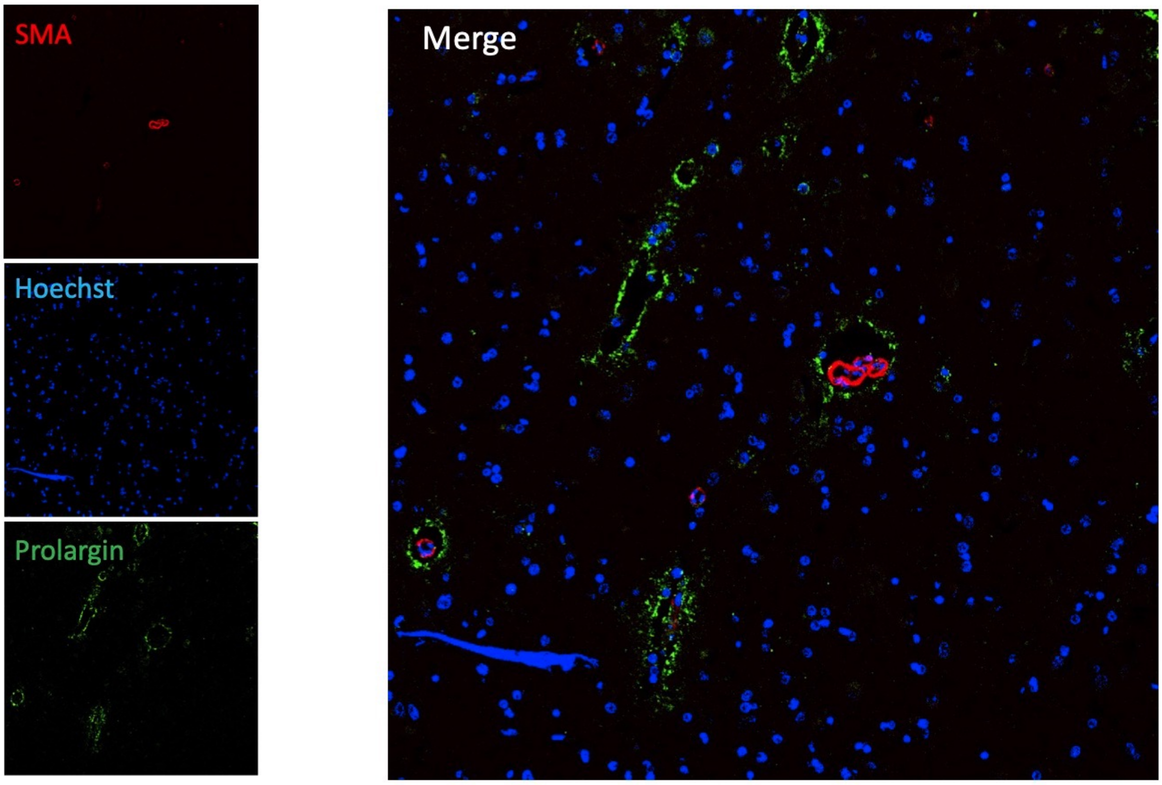

Supplementary Material Bibliography:

1. Fischer R, Trudgian DC, Wright C, et al. Discovery of candidate serum proteomic and metabolomic biomarkers in ankylosing spondylitis. *Mol Cell Proteomics MCP*. 2012;11(2):M111.013904. doi:10.1074/mcp.M111.013904

2. Gil-Dones F, Alonso-Orgaz S, Avila G, et al. An optimal protocol to analyze the rat spinal cord proteome. *Biomark Insights*. 2009;4:135-164. doi:10.4137/bmi.s2965

3. Tyanova S, Temu T, Sinitcyn P, et al. The Perseus computational platform for comprehensive analysis of (prote)omics data. *Nat Methods*. 2016;13(9):731-740. doi:10.1038/nmeth.3901

4. Pisa M, Pansieri J, Yee S, et al. Anterior optic pathway pathology in CNS demyelinating diseases. *Brain J Neurol*. 2022;145(12):4308-4319. doi:10.1093/brain/awac030

5. Schindelin J, Arganda-Carreras I, Frise E, et al. Fiji - an Open Source platform for biological image analysis. *Nat Methods*. 2012;9(7):10.1038/nmeth.2019. doi:10.1038/nmeth.2019

6. Kuhlmann T, Ludwin S, Prat A, Antel J, Brück W, Lassmann H. An updated histological classification system for multiple sclerosis lesions. *Acta Neuropathol (Berl)*. 2017;133(1):13-24. doi:10.1007/s00401-016-1653-y

7. DeLuca GC, Alterman R, Martin JL, et al. Casting light on multiple sclerosis heterogeneity: the role of HLA-DRB1 on spinal cord pathology. *Brain J Neurol*. 2013;136(Pt 4):1025-1034. doi:10.1093/brain/awt031
